# Genome-wide association study of cardiovascular disease in testicular cancer patients treated with platinum-based chemotherapy

**DOI:** 10.1038/s41397-020-00191-8

**Published:** 2020-10-03

**Authors:** Lars C. Steggink, Hink Boer, Coby Meijer, Joop D. Lefrandt, Leon W. M. M. Terstappen, Rudolf S. N. Fehrmann, Jourik A. Gietema

**Affiliations:** 1 Department of Medical Oncology, University Medical Center Groningen, University of Groningen, Groningen, Hanzeplein 1, 9713 GZ Groningen, The Netherlands; 2 Department of Internal Medicine, Division of Vascular Medicine, University Medical Center Groningen, University of Groningen, Groningen, Hanzeplein 1, 9713 GZ Groningen, The Netherlands; 3 Medical Cell BioPhysics, University of Twente, Drienerlolaan 5, 7522 NB Enschede, The Netherlands

**Keywords:** Medical research, Outcomes research, Medical research, Outcomes research

## Abstract

Genetic variation may mediate the increased risk of cardiovascular disease (CVD) in chemotherapy-treated testicular cancer (TC) patients compared to the general population. Involved single nucleotide polymorphisms (SNPs) might differ from known CVD-associated SNPs in the general population. We performed an explorative genome-wide association study (GWAS) in TC patients. TC patients treated with platinum-based chemotherapy between 1977 and 2011, age ≤55 years at diagnosis, and ≥3 years relapse-free follow-up were genotyped. Association between SNPs and CVD occurrence during treatment or follow-up was analyzed. Data-driven Expression Prioritized Integration for Complex Trait (DEPICT) provided insight into enriched gene sets, i.e., biological themes. During a median follow-up of 11 years (range 3–37), CVD occurred in 53 (14%) of 375 genotyped patients. Based on 179 SNPs associated at *p* ≤ 0.001, 141 independent genomic loci associated with CVD occurrence. Subsequent, DEPICT found ten biological themes, with the RAC2/RAC3 network (linked to endothelial activation) as the most prominent theme. Biology of this network was illustrated in a TC cohort (*n* = 60) by increased circulating endothelial cells during chemotherapy. In conclusion, the ten observed biological themes highlight possible pathways involved in CVD in chemotherapy-treated TC patients. Insight in the genetic susceptibility to CVD in TC patients can aid future intervention strategies.

## Introduction

Testicular cancer (TC) is the most common malignancy in men between 20 and 40 years of age. Over 80% of patients with metastatic TC is cured with platinum-based chemotherapy [1]. Consequently, the number of TC survivors steadily increases. High cure rates come at the trade-off of increased risk of cardiovascular disease (CVD), attributed to chemotherapy. Compared to age-matched controls, patients treated with bleomycin, etoposide, and cisplatin chemotherapy have a hazard ratio for coronary artery disease of 5.7 (95% confidence interval, CI, 1.9–17.1), and for atherosclerotic disease of 4.7 (95% CI, 1.8–12.2) [2]. Moreover, cardiovascular mortality is increased after chemotherapy for TC with a standardized mortality ratio of 1.36 (95% CI, 1.03–1.78) [3]. In these relatively young men, the burden of cardiovascular morbidity and mortality due to chemotherapy is an important determinant of long-term outcome.

Genetic variation is thought to mediate in part the increased risk of CVD in chemotherapy-treated TC patients. However, involved single nucleotide polymorphisms (SNPs) might differ from the currently known SNPs associated with increased risk of CVD in the general population.

A limited number of studies reported on associations between SNPs and late complications of TC treatment. These studies mostly focused on SNPs in specific genes rather than using unbiased genome-wide approaches. SNPs in the glutathione S-transferase genes GSTP1 and GSTM3 have been associated with neurotoxicity and ototoxicity in platinum-treated TC patients [4–6]. SNPs in the 5-α-reductase type II (SRD5A2) gene were associated with prevalence of the metabolic syndrome in TC survivors after platinum-based chemotherapy [7]. This finding, however, could not be replicated [8]. A recent study in 188 platinum-treated TC patients reported that a SNP in solute carrier gene SLC16A5 was associated with cisplatin-induced ototoxicity [9]. In the Platinum Study cohort of 511 TC survivors, a SNP in the Wolframin ER transmembrane glycoprotein (WFS1) was associated with ototoxicity [10].

To date, however, genetic variation has not been investigated in relation to CVD after TC treatment. As with the reported associations between SNPs and neurotoxicity, ototoxicity, and the metabolic syndrome, insight in genetic variation and biological pathways associated with cardiovascular toxicity may help to estimate the increased risk for CVD and to guide preventive cardiovascular intervention strategies in TC patients treated with platinum-based chemotherapy. We performed an exploratory genome-wide association study (GWAS) in TC patients treated with platinum-based chemotherapy to gain insight into the SNPs underlying susceptibility to CVD in this population.

## Patients and methods

### Patient selection and phenotype data

Patients were selected from the institutional data-biobank on TC patients treated at the University Medical Center Groningen between 1977 and 2011. Inclusion criteria were (a) advanced seminoma or non-seminoma TC, Royal Marsden Hospital stage II, III, or IV, (b) treated with platinum-based chemotherapy, (c) age ≤55 years at diagnosis, (d) ≥3 years relapse-free follow-up after start of first-line chemotherapy, or after second-line chemotherapy if given for early relapse within 3 years after TC diagnosis, (e) no chemotherapy or malignancy prior to TC, and (f) no CVD prior to TC.

The phenotype endpoint of the GWAS was the occurrence of a cardiovascular event during treatment or follow-up, defined as any (1) coronary disease (myocardial infarction, acute coronary syndrome), (2) cerebrovascular infarction or transient ischemic attack, (3) cardiomyopathy or heart failure, (4) thromboembolic event (deep venous thrombosis, pulmonary embolism, venous access port-associated), (5) peripheral artery disease, or (6) other cardiovascular events (e.g., intracerebral hemorrhage, cardiac arrhythmia, or cardiac valve regurgitation or stenosis). Follow-up data were available through several prospective TC studies and medical records. Follow-up was censored in case of late relapse (more than 3 years after chemotherapy), except for teratoma treated by surgery alone. In addition, follow-up was censored in case of second malignancy, except for non-melanoma skin cancer. Study protocols were approved by the medical ethical review committee of the University Medical Center Groningen (ethical protocol code 2006/041), performed in accordance with the Declaration of Helsinki and each participant gave written informed consent.

### Genotype data

Germline DNA was isolated from blood using a standard phenol-chloroform method or using the NucleoSpin Blood XL column (Macherey-Nagel, BIOKÉ, Leiden, The Netherlands). SNP array was performed according to suppliers’ protocol using an Illumina HumanCytoSNP-12 v2.1 BeadChip (Illumina, San Diego, California, US) covering 298,563 SNPs. SNPs were called using Illumina GenomeStudio v2011.1 (Genotyping v1.9.4; Illumina Genome Viewer v1.9.0) and then exported (PLINK Input Report Plug-in v2.1.3 for GenomeStudio Software).

### Genotyping quality analysis

Quality analysis was performed using PLINK 1.07, filtering out (1) SNPs with a call rate <95%, (2) samples with a SNP call rate <95%, (3) samples with mismatch between reported and predicted sex, (4) SNPs that have minor allele frequency <5%, and (5) samples with significant deviation from the Hardy–Weinberg equilibrium (*p* < 0.0001) [11].

### Association analysis

Association between SNPs and the occurrence of CVD was assessed in PLINK by chi-squared test using the max(T) permutation procedure with 25,000 permutations. SNPs were clumped into independently associated loci based on linkage disequilibrium, using an empirical *p* ≤ 0.001 as a threshold for association of index SNPs (PLINK parameters: --clump-p1 0.001 --clump-p2 0.01 --clump-r2 0.20 --clump-kb 500).

### Annotation of associated SNPs: genes

The loci found in the association analysis were annotated with genes known to be associated with these loci using the following three methods. First, we determined the nearest gene(s) for each SNP as reported in dbSNP build 150. Second, based on a publicly available large-scale mapping of *cis* and *trans* expression quantitative trait loci (eQTLs) in blood we determined for each SNP if that SNP has been reported to affect the expression of any gene [12]. Third, the SNPs found in the association analysis were used as input for the Data-driven Expression Prioritized Integration for Complex Trait (DEPICT) framework [13]. At the core of DEPICT, genes are functionally characterized by their membership probabilities across 14,461 gene sets. DEPICT takes genes in loci associated with the input SNPs and uses the shared gene set memberships of those genes to prioritize genes that have similar predicted functions. Genes were reported by HGNC gene symbol if possible (retrieved using R/Bioconductor package biomaRt 2.32.1), or alternatively by Ensemble identifiers.

### Insight in associated SNPs: gene sets clustered into ‘biological themes’

Next, gene set enrichment analysis was performed to gain insight in the biology underlying the associations between SNPs and the occurrence of CVD. To this end, DEPICT takes genes in loci associated with the input SNPs, and tests which of its 14,461 gene sets are enriched in those genes, at a threshold of nominal *p* < 0.001. Since DEPICT is based on reconstituted gene sets of known molecular pathways from various sources, overlap between the reconstituted gene sets can be expected. Therefore, the enriched gene sets were clustered into ‘biological themes’ using affinity propagation as described next.

The enriched gene sets were extracted from DEPICT resource file containing the gene-gene set matrix of *z*-scores for 19,987 genes. Next, pairwise Pearson correlation coefficients were computed between all enriched gene sets, and similar gene sets were clustered into biological themes using affinity propagation clustering (using R package apcluster 1.4.4) [14]. Affinity propagation finds exemplar gene sets within the input gene sets that are representative for each of the clusters, and names the clusters after their exemplar gene set. As stated by the authors of DEPICT, the reconstituted gene sets should be interpreted in light of the genes that are mapped to them, since their identifiers are simply carried over from the predefined gene sets used in the development of DEPICT [13]. For the biological themes, we addressed this issue by renaming the biological theme, if necessary, after examining the main genes within each biological theme (determined by the absolute weighted mean *z*-score for each gene in the biological theme, using the multiplicative inverse of the nominal p of each gene set as a weight for each *z*-score).

### Circulating endothelial cells during chemotherapy as indicator of endothelial activation

As a measure of cancer treatment-induced endothelial activation, the number of circulating endothelial cells (CECs) were measured in 60 patients with metastatic TC before and during three consecutive cycles of chemotherapy with bleomycin, etoposide, and cisplatin (BEP) using the CellSearch CEC Kit (Menarini Silicon Biosystems, Huntingdon Valley, PA, USA) according to the supplier’s protocol [15]. In short, blood was collected in Cell Save Preservative tubes, and CECs were immunomagnetically enriched targeting CD146 followed by staining of the enriched cell population for CD45, CD105, and DAPI. The CD146 enriched cells were classified as CECs when CD105+/CD45−DAPI+ cells.

## Results

**Fig. 1:**
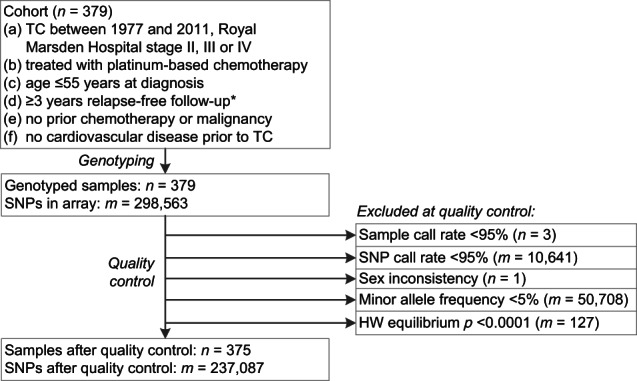
Patient selection and quality control. * ≥3 years relapse-free follow-up after start of first-line chemotherapy, or after second-line chemotherapy if given for early relapse within 3 years after TC diagnosis. HW equilibrium: Hardy–Weinberg equilibrium.

Of the 379 genotyped patients, genotypes of 375 TC patients and 237,087 SNPs passed quality analysis (Fig. 1). All 375 TC patients had been treated with platinum-based chemotherapy between 1977 and 2011 (Table 1). Most chemotherapy regimens also contained bleomycin (*n* = 356, 95%). During a median follow-up of 11 years (range 3–37), CVD had occurred in 53 cases (14%). These cases were median 4 years older at start of chemotherapy than patients who had no CVD (*p* = 0.009). At follow-up, cases met the criteria for metabolic syndrome more often (67% versus 37%) and used more antihypertensive and lipid lowering drugs than controls (*p* < 0.001 for all).

**Table 1:** Baseline patient and treatment characteristics and clinical follow-up data (*n* = 375).

	Cases (*n* = 53)		Controls (*n* = 322)		*p*
	Median (range) or *n* (%)	Missing *n* (%)	Median (range) or *n* (%)	Missing *n* (%)	
Age at start chemotherapy (years)	31 (17–55)	–	27 (16–55)	–	0.009
Age at follow-up (years)	51 (24–72)	–	41 (21–69)	–	<0.001
Follow-up duration (years)	12 (4–37)	–	11 (3–37)	–	0.03
Royal Marsden Hospital stage		–		3 (1%)	0.46
Stage II	25 (47%)		179 (56%)		
Stage III	6 (11%)		31 (10%)		
Stage IV	22 (42%)		109 (34%)		
IGCCCG classification		2 (4%)		3 (1%)	0.95
Good	33 (62%)		197 (61%)		
Intermediate	14 (26%)		90 (28%)		
Poor	4 (8%)		32 (10%)		
Chemotherapy regime		–		–	–
BEP followed by EP	18 (33%)		129 (40%)		
BEP	17 (32%)		114 (35%)		
PVB followed by PV maintenance	3 (6%)		16 (5%)		
2 PVB followed by 2 BEP	1 (2%)		16 (5%)		
PVB	4 (8%)		11 (3%)		
EP	5 (9%)		8 (2%)		
CEB	0		10 (3%)		
Other platinum-based chemotherapy	5 (9%)		18 (6%)		
Any radiotherapy	4 (8%)	–	4 (1%)	–	–
Abdominal	2 (4%)		2 (0.6%)		
Cranial	0		2 (0.6%)		
Thoracic	1 (2%)		0		
Contralateral testicle	1 (2%)		0		
No radiotherapy	49 (93%)		318 (99%)		
Cardiovascular events		–		–	–
Any event	53 (100%)		0		
Cardiomyopathy^a^	6 (11%)		–		
Cerebrovascular^a^	8 (15%)		–		
Coronary^a^	19 (36%)		–		
Thromboembolic^a^	19 (36%)		–		
Other^b^	5 (9%)		–		
None	0		322 (100%)		
Blood pressure at follow-up (mmHg)		3 (6%)		26 (8%)	
Systolic	139 (110–190)		135 (105–190)		0.07
Diastolic	85 (60–112)		80 (50–124)		0.07
BMI at follow-up (kg/m²)	26.4 (19.4–40.6)	5 (9%)	25.5 (19.3–41.7)	58 (18%)	0.04
Waist-hip ratio at follow-up	1.0 (0.9–1.3)	8 (15%)	1.0 (0.8–1.3)	95 (30%)	0.60
Metabolic syndrome at follow-up		5 (9%)		75 (23%)	<0.001
Yes	32 (60%)		92 (29%)		
No	16 (30%)		155 (48%)		
Antihypertensive drugs at follow-up		–		4 (1%)	<0.001
Yes	27 (51%)		52 (16%)		
No	26 (49%)		266 (83%)		
Lipid lowering drugs at follow-up		–		3 (1%)	<0.001
Yes	21 (40%)		27 (8%)		
No	32 (60%)		292 (91%)		
Antidiabetic drugs at follow-up		–		3 (1%)	0.06
Yes	4 (8%)		7 (2%)		
No	49 (93%)		312 (97%)		
Testosterone suppletion at follow-up		–		3 (1%)	0.75
Yes	2 (4%)		20 (6%)		
No	51 (96%)		299 (93%)		
Fasting glucose at follow-up (mmol/l)	5.6 (3.5–15.1)	3 (6%)	5.4 (1.3–9.4)	104 (32%)	0.05
Total cholesterol at follow-up (mmol/l)	4.6 (3.1–7.6)	–	5.2 (2.8–9.7)	5 (2%)	<0.001
HDL cholesterol at follow-up (mmol/l)	1.4 (0.8–4.9)	6 (11%)	1.2 (0.3–5.6)	56 (17%)	0.17
LDL cholesterol at follow-up (mmol/l)	2.9 (1.2–5.8)	6 (11%)	3.3 (0.4–6.5)	57 (17%)	0.03
Triglyceride at follow-up (mmol/l)	1.4 (0.5–4.7)	–	1.4 (0.4–118)	4 (1%)	0.90
Total testosterone at follow-up (nmol/l)	13 (4.7–33)	6 (11%)	15 (0.7–160)	57 (18%)	0.01
eGFR at follow-up (ml/min/1.73 m²)	90 (18–127)	–	92 (18–127)	4 (1%)	0.02
Albuminuria in 24 h urine at follow-up (mg/24 h)	6 (0.9–1,152)	33 (62%)	8 (0–5,850)	158 (49%)	–
Chronic kidney disease stage at follow-up		–		4 (1%)	0.01
Stage 4	0		1 (0.3%)		
Stage 3B	3 (6%)		1 (0.3%)		
Stage 3A	2 (4%)		12 (4%)		
Stage 2	26 (49%)		130 (40%)		
Stage 1	22 (42%)		174 (54%)		

Characteristics of TC patients with and without cardiovascular event were compared with Fisher’s exact test and Mann–Whitney U test after removal of missing values, with two-sided *p* < 0.05 considered significant.

*BEP* bleomycin, etoposide, cisplatin, *BMI* body-mass index, *CEB* carboplatin, etoposide, bleomycin, *eGFR* estimated glomerular filtration rate, *EP* etoposide, cisplatin, *HDL* high density lipoprotein, *IGCCCG* International Germ Cell Cancer Collaborative Group, *LDL* low-density lipoprotein, *PV* cisplatin, vinblastine, *PVB* cisplatin, vinblastine, bleomycin.

^a^Patients with multiple cardiovascular events were counted in multiple categories.

^b^Intracerebral hemorrhage (*n* = 1), cardiac arrhythmia (*n* = 2), and cardiac valve regurgitation or stenosis (*n* = 2).

In total, 179 SNPs were associated at *p* ≤ 0.001 with the occurrence of any CVD during or after chemotherapy (Table S1). Clumping based on linkage disequilibrium resulted in 141 independent loci containing a total of 324 SNPs. These 141 loci and corresponding SNPs were annotated by finding nearest gene(s), *cis* or *trans* eQTLs, or DEPICT gene prioritization (Table 2). Since DEPICT only includes autosomal loci that do not overlap with the major histocompatibility complex region, gene prioritization resulted in 187 genes mapped to 129 loci.

**Table 2:** For the 141 identified candidate loci, related genes were found by proximity, *cis* or *trans* eQTLs, or by gene prioritization in DEPICT.

Locus	SNPs	*p*	Genes
1	rs983098(*), rs1374038, rs10461655	0.00004	PARP8(d)
2	rs1352436(*)	0.00004	ENSG00000250546(d)
3	rs12692720(*), rs6432774, rs1528431, rs6743187	0.00004	RND3(n,d), LINC01920(d)
4	rs199635(*), rs852937, rs543827, rs473757, rs6918162	0.00004	LINC01626(n), LINC00472(d), OGFRL1(ce)
5	rs34814294(*)	0.00004	MMP28(n*,d), CCL5(d), HEATR9(d), RDM1(d), TAF15(d), LYZL6(d)
6	rs3849324(*)	0.00008	MALL(n*,d), LINC00116(d), NPHP1(d)
7	rs6538046(*), rs10858436, rs4503615, rs2406250, rs2406254	0.00008	MGAT4C(n*,d)
8	rs4755718(*), rs7929359, rs12273774, rs4755689, rs7929102, rs10768008, rs7939586	0.00008	KIAA1549L(n*,d), ENSG00000255207(d)
9	rs11874286(*), rs1025206, rs16970618, rs12457667, rs8093155	0.00008	LOC105372076(n#), MIR924HG(d)
10	rs6687976(*),	0.00012	LINC01676(d)
11	rs2123269(*), rs2100346, rs6987013	0.00012	MRPS28(n*,d), TPD52(d,ce)
12	rs7744306(*), rs9347666, rs9456798, rs10945861	0.00012	PACRG(n*,d), PARK2(n,d)
13	rs10950657(*), rs6968554, rs1476080	0.00012	AHR(n,d), LOC101927609(n#), ENSG00000237773(d#), ENSG00000236318(d#)
14	rs9459964(*)	0.00016	LOC105378150(n*#), ENSG00000232197(d#)
15	rs4466027(*), rs7673254, rs13121254	0.00016	LINC02261(n*), STIM2(d)
16	rs6988639(*), rs1433393, rs2656118	0.00016	SNTB1(d), HAS2(d)
17	rs12439991(*), rs7163517, rs11857756	0.00016	LOC105370777(n*), ENSG00000259450(d)
18	rs10932020(*), rs7591187	0.00016	CD28(d,ce)
19	rs6813846(*), rs9997501, rs892836	0.0002	STOX2(n*,d)
20	rs7748814(*), rs7742883, rs6914805, rs6459467	0.0002	GMPR(d,ce), ATXN1(d,ce)
21	rs2331545(*)	0.0002	OVAAL(n*,d)
22	rs676740(*)	0.0002	AFDN(n*,d), ENSG00000235994(d)
23	rs1263635(*), rs943888	0.00024	TRAC(d)
24	rs755535(*), rs12108497, rs2130392, rs4069938	0.00024	PRIMPOL(n,d,ce), CENPU(n,d,ce), ACSL1(n), CASP3(d,ce)
25	rs11164896(*), rs2783499	0.00024	CCDC18(n,d,ce), DR1(d,ce), TMED5(d,ce), MTF2(d), FNBP1L(d), CCDC18-AS1(d)
26	rs9324446(*), rs3887806, rs7837472	0.00024	FAM135B(d)
27	rs3934720(*)	0.00024	EIF2B5(d)
28	rs7702793(*)	0.00024	LOC105379160(n*#), GRAMD3(d)
29	rs10034996(*)	0.00024	ENSG00000251199(d)
30	rs1826613(*), rs1227842	0.00024	DLG2(n*,d)
31	rs4757245(*), rs4756786, rs2970335, rs11023194, rs11023197, rs6486191, rs11023210, rs3923294, rs12295888, rs11023223, rs2575825, rs10832275	0.00028	SPON1(n,d), PDE3B(d), RRAS2(d), COPB1(d,ce)
32	rs1387092(*), rs1488745, rs6802020	0.00028	CNTN4(d)
33	rs17790008(*), rs6584652	0.00032	SORCS3(n*,d)
34	rs10828065(*), rs12355916	0.00032	PLXDC2(d)
35	rs596557(*)	0.00032	TMX3(d)
36	rs17377955(*)	0.00032	DGKB(d)
37	rs11582429(*), rs2168951	0.00032	LINC01732(d)
38	rs9949956(*), rs8095771, rs12966370, rs7236288, rs10871565	0.00032	LINC01029(n), GALR1(d)
39	rs6816525(*)	0.00032	IRF2(n*,d)
40	rs4896501(*), rs7753475	0.00032	CLVS2(d)
41	rs2554728(*)	0.00032	CSMD1(n*,d)
42	rs10503759(*), rs969456	0.00032	LOC101929294(n*#), ADAM7(d), ENSG00000253643(d#), ADAMDEC1(ce)
43	rs4858795(*), rs6794875	0.00032	SHISA5(n*,d), PLXNB1(n,d), CCDC51(d), FBXW12(d), PFKFB4(d,ce), TREX1(ce), ATRIP(ce), NME6(ce), NCKIPSD(ce)
44	rs4662553(*)	0.00036	LRP1B(d)
45	rs17756443(*), rs16959991	0.00036	CDH13(n*,d)
46	rs5931289(*), rs5929883, rs5931353	0.00036	–
47	rs12920637(*), rs7198542	0.00036	CDH13(n*,d)
48	rs7745485(*), rs12524966, rs12198618	0.00036	LOC105374974(n#), HDGFL1(d)
49	rs6692(*), rs9555784	0.0004	ARHGEF7(n*,d,ce)
50	rs6467607(*)	0.0004	SLC13A4(d)
51	rs10902531(*)	0.0004	SFSWAP(d)
52	rs2215375(*)	0.0004	SPP2(d)
53	rs7101204(*)	0.0004	SVIL(n*,d)
54	rs1555145(*)	0.0004	BTBD3(d)
55	rs13243936(*)	0.0004	EPDR1(d), NME8(ce), GPR141(ce)
56	rs11772261(*), rs11770352, rs6462776, rs4723679, rs6462780	0.0004	EPDR1(d)
57	rs4432837(*), rs6864394	0.0004	RGS7BP(n*,d)
58	rs11736162(*), rs1439381, rs1439382, rs7684647	0.00044	GUF1(d,ce), GNPDA2(ce)
59	rs9459963(*), rs9366130, rs4540249	0.00044	ENSG00000232197(d), DLL1(ce), FAM120B(ce)
60	rs4676617(*)	0.00044	LOC102724104(n*#), CX3CR1(d,ce), WDR48(ce)
61	rs11025878(*), rs4644637, rs7941875	0.00044	NELL1(n*,d)
62	rs2040664(*)	0.00044	DNAH11(n*,d)
63	rs11066610(*), rs16942882, rs11066638	0.00044	LHX5(d), LINC01234(d)
64	rs1366906(*), rs6485532	0.00048	CD82(d)
65	rs3866223(*), rs11241999	0.00048	ADAMTS19(n*,d)
66	rs2275696(*), rs3892248	0.00048	NFASC(n*,d), DSTYK(ce)
67	rs12688573(*), rs5936239, rs2341921, rs758439	0.00048	AFF2(n*)
68	rs2027469(*)	0.00048	CRP(d), DUSP23(ce)
69	rs11800877(*), rs4657327, rs1415439, rs12076657	0.00048	PBX1(d)
70	rs2070584(*)	0.00052	TIMP1(n*)
71	rs28890299(*)	0.00052	LIPI(n*)
72	rs11643432(*), rs10514583	0.00052	CDH13(n*,d)
73	rs4237648(*), rs10742717, rs4237647, rs1665150	0.00052	TSPAN18(n,d)
74	rs4689203(*), rs13123841, rs10937629, rs13121492	0.00056	STK32B(n*,d)
75	rs9530423(*), rs1359500, rs9573531	0.00056	TBC1D4(n*,d)
76	rs7143719(*),	0.00056	TSHR(n*,d)
77	rs10822863(*), rs2894011, rs2894015, rs4143863	0.00056	CTNNA3(n*,d)
78	rs7610664(*), rs7609933	0.00056	FGF12(n*,d)
79	rs6558831(*), rs12680491, rs11136689	0.00056	CSMD1(n*,d)
80	rs4751878(*), rs4752666, rs10887101, rs6585804, rs11592039	0.00056	TACC2(n*,d)
81	rs12965155(*)	0.00056	MIR924HG(d)
82	rs760150(*)	0.00056	PCP4(n*,d), TMPRSS3(d)
83	rs239953(*)	0.00056	POR(n*,d,ce), RHBDD2(d,ce)
84	rs10182928(*)	0.00056	SATB2(d), SATB2-AS1(d)
85	rs617459(*), rs657426	0.0006	SETBP1(n*,d)
86	rs12165104(*), rs12954590	0.0006	TNFRSF11A(n*,d), ZCCHC2(d)
87	rs11688528(*)	0.00064	LOC100506474(n*#), TRIB2(d)
88	rs6966799(*)	0.00064	HDAC9(d)
89	rs6759648(*), rs7593846, rs9941639	0.00064	LINC01798(n*), MEIS1(d)
90	rs2973419(*)	0.00064	PRR16(d)
91	rs10456118(*), rs10948172, rs857601, rs3799977, rs4714828, rs10948197, rs6919813	0.00064	SUPT3H(n,d,ce), RUNX2(d)
92	rs4795934(*), rs990510, rs12944367	0.00064	TMEM132E(d)
93	rs7831168(*), rs13269649, rs907991	0.00068	FAM135B(d)
94	rs9880546(*)	0.00068	LINC00578(n*), TBL1XR1(d)
95	rs1375547(*), rs9861237, rs9822731, rs12498010, rs9880919	0.00068	CADM2(n*,d)
96	rs6557678(*), rs6988938, rs7824718, rs7009973, rs7008867	0.00068	SLC25A37(d,ce), ENTPD4(ce), AC051642.5(ce#)
97	rs16993897(*)	0.00068	VAV1(d), ADGRE1(ce)
98	rs16999330(*), rs4434196	0.00068	FSTL5(n*,d)
99	rs16880318(*), rs16880352	0.00072	KCNV1(d)
100	rs4947522(*), rs28633916, rs9642409	0.00072	COBL(d)
101	rs17078840(*)	0.00072	LINC00327(d)
102	rs40566(*)	0.00072	C5orf67(n*), MAP3K1(d)
103	rs4528743(*)	0.00072	SLC16A14(n*,d)
104	rs13249135(*)	0.00072	MIR2052HG(n*,d)
105	rs747925(*), rs11236683	0.00072	LOC105369395(n*#), THAP12(d)
106	rs10505371(*), rs17803964	0.00076	ENPP2(n*,d), TAF2(ce)
107	rs3911618(*)	0.00076	RGS7(n*,d)
108	rs7691972(*)	0.00076	ACSL1(n*,d), CASP3(ce)
109	rs6507498(*), rs9807753, rs8093542	0.00076	CABLES1(d)
110	rs7722584(*), rs11948927	0.00076	NLN(d)
111	rs3812278(*), rs10255837	0.00076	CNOT4(n*,d), NUP205(d)
112	rs6773957(*), rs6444175	0.0008	ADIPOQ(n*,d), LYST(te)
113	rs10858680(*), rs10777082, rs11104704, rs11104713	0.0008	C12orf50(n*,d)
114	rs4577099(*)	0.0008	LOC102724084(n*#), DYNLRB2(d)
115	rs8030490(*)	0.0008	AKAP13(n*,d,ce)
116	rs11610234(*), rs4334084	0.0008	TMEM132B(n*,d)
117	rs4762060(*)	0.0008	KRT80(n*,d), C12orf80(d)
118	rs6673313(*)	0.0008	LOC105378764(n*#), NFIA(d)
119	rs2506145(*)	0.00084	NRP1(n*,d)
120	rs898918(*), rs12100703	0.00084	LINC01550(d)
121	rs4607409(*), rs300121, rs777573, rs9356411, rs9348092	0.00084	LINC00473(n,d), LOC105378117(n#), T(d), MPC1(ce)
122	rs2147866(*), rs6891675	0.00084	CCDC192(n*), LINC01184(d)
123	rs8178838(*)	0.00088	APOH(n*,d), CEP112(d)
124	rs13406850(*), rs10172452, rs1628975	0.00088	LRP1B(d)
125	rs745247(*), rs7739748	0.00092	CD83(d)
126	rs6934819(*)	0.00092	ENPP3(d)
127	rs7916162(*)	0.00092	TACC2(n*,d), PLEKHA1(ce)
128	rs10764344(*), rs11013053	0.00092	PIP4K2A(n*,d), PIP5K2A(ce)
129	rs3003177(*)	0.00092	ENSG00000223786(d#)
130	rs949719(*), rs1516651	0.00092	ATP10B(n*,d)
131	rs938025(*)	0.00096	LINC00616(d), SLC7A11-AS1(d)
132	rs11640395(*)	0.00096	ZFHX3(n*,d)
133	rs2201369(*), rs10481102, rs13439041	0.00096	BAALC(n*,d), BAALC-AS2(d)
134	rs4521178(*)	0.00096	CPB1(n*,d), CPA3(d,ce)
135	rs1400438(*), rs1516893	0.00096	LINC01505(d)
136	rs9864293(*)	0.00096	IL1RAP(d)
137	rs2236570(*), rs613089	0.00096	BCL9(n*,d,ce), ACP6(d)
138	rs10868152(*), rs7022329	0.00096	SLC28A3(n*,d)
139	rs10501827(*)	0.001	SESN3(d)
140	rs8064765(*), rs11656652, rs11079045, rs1032070, rs8069972, rs2292755, rs4792992, rs4793253, rs7224577	0.001	ATP6V0A1(n,d), CAVIN1(n,d), LOC102725238(n#), EZH1(d), CCR10(d), PLEKHH3(d), RETREG3(n,d), MLX(n,d), TUBG1(d), COASY(d,ce), CNTNAP1(d), TUBG2(d), HSD17B1(d), NAGLU(d), PSMC3IP(d), STAT3(ce), BECN1(ce)
141	rs12750904(*)	0.001	ABCD3(n*,d,ce), F3(d)

Loci are ranked by *p*, which is the empirical *p* for association of the index SNP with cardiovascular events. Reference SNP cluster IDs are reported for the index SNP (marked with an asterisk) and non-index clumped SNPs in the locus. For all SNPs in the locus, relevant genes were identified by proximity to the index or non-index SNPs (annotated with n in parenthesis, with an asterisk denoting genes in proximity to the index SNP). Additional relevant genes are reported based on *cis* and *trans* eQTLs (annotated with ce and te, respectively), and based on gene prioritization in DEPICT (annotated with d). Genes are reported by HGNC gene symbol if possible, or alternatively by Ensemble identifiers if no HGNC gene symbol exists.

*DEPICT* data-driven expression-prioritized integration for complex traits, *eQTL* expression quantitative trait loci, *HGNC* human genome organisation gene nomenclature committee, *SNP* single nucleotide polymorphism.

**Fig. 2:**
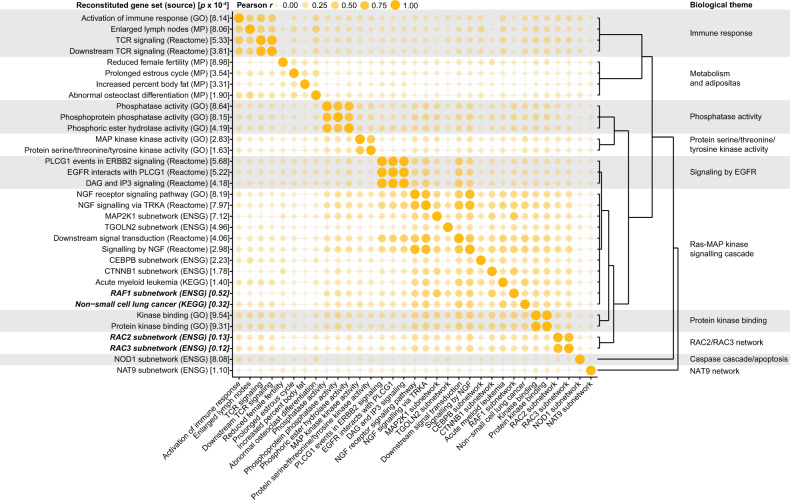
The 33 gene sets that were enriched according to DEPICT were clustered into ten biological themes. The reconstituted gene sets were named after the predefined gene sets used in the development of DEPICT, and source databases are reported in brackets. The nominal enrichment *p* for each reconstituted gene set as reported by DEPICT is reported in brackets, with emphasis added for gene sets with *p* < 1 × 10^−4^. Clustering into biological themes was performed using affinity propagation clustering after calculating pairwise Pearson correlation between all enriched gene sets, as depicted in the bubble chart. CEBPB: CCAAT/enhancer binding protein beta, CTNNB1: catenin beta 1, DAG: diacylglycerol, EGFR: epidermal growth factor receptor, ENSG: Ensembl gene, ERBB2: erb-b2 receptor tyrosine kinase 2, GO: gene ontology, IP3: inositol triphosphate, KEGG: Kyoto Encyclopedia of Genes and Genomes, MAP: mitogen-activated protein kinase, MAP2K1: MAP kinase kinase 1, MP: mammalian phenotype ontology, NAT9: N-acetyltransferase 9, NGF: nerve growth factor, NOD1: nucleotide binding oligomerization domain containing 1, PLCG1: phospholipase C, gamma 1, RAC2: ras-related C3 botulinum toxin substrate 2, RAC3: ras-related C3 botulinum toxin substrate 3, RAF1: Raf-1 proto-oncogene, serine/threonine kinase, TCR: T cell receptor; TGOLN2: trans-golgi network protein 2, TRKA: tropomyosin receptor kinase A.

Next, gene set enrichment analysis was performed in DEPICT to gain insight in the biology underlying the associations between SNPs and CVD, resulting in 33 gene sets (*p* < 0.001, Table S2). These 33 gene sets were subsequently clustered into ten distinct gene set clusters to highlight the biological themes that are underlying the associations between SNPs and CVD in chemotherapy-treated TC patients (Fig. 2 and Table S3). These biological themes included the RAC2/RAC3 network, metabolism and adiposity, immune response, and caspase cascade/apoptosis.

**Fig. 3:**
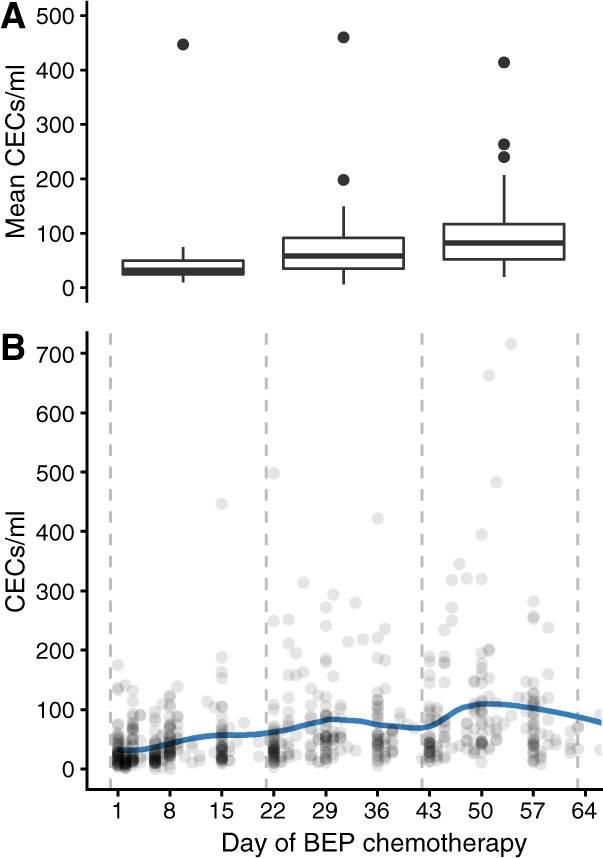
The number of CECs rises during three cycles of BEP chemotherapy, based on measurements in 665 blood samples in 60 TC patients. Depicted are (**a**) the mean number of CECs per patient during each cycle, and (**b**) all CEC measurements with a LOESS curve. During the three consecutive cycles of chemotherapy the mean number of CECs per patient were median 32/ml (interquartile range, IQR 24–50), 58/ml (IQR 35–91), and 82/ml (20–117), respectively. Compared to the first cycle, the mean number of CECs was increased in the second and third cycle of chemotherapy (Wilcoxon signed rank test *p* < 0.001). BEP: bleomycin, etoposide, cisplatin, CEC: circulating endothelial cell.

Since the most enriched gene sets were the RAC2 and RAC3 subnetworks, clustered as the RAC2/RAC3 network, we explored a biological readout of this finding. RAC2 and RAC3 have been implicated in endothelial activation and dysfunction [16, 17]. Therefore, as a measure of endothelial activation, the number of CECs were measured over time in 60 patients with metastatic TC treated with chemotherapy. A significant increase in CECs during three consecutive cycles of platinum-based chemotherapy was observed (Fig. 3).

## Discussion

In this explorative GWAS using a contemporary strategy in TC patients treated with platinum-based chemotherapy, we determined which SNPs were associated with the occurrence of CVD after start of chemotherapy in these patients. Subsequent gene set enrichment analysis resulted in ten biological themes that highlight pathways that may be involved in the occurrence of CVD in chemotherapy-treated TC patients. These themes include the RAC2/RAC3 network, metabolism and adiposity, immune response, and caspase cascade/apoptosis.

Of special interest is the RAC2/RAC3 network that was identified as a prominent biological theme. Several recent reports link RAC2 and RAC3 to chemotherapy toxicity. Rac2 deficiency protected Rac2−/− mice from bleomycin-induced pulmonary fibrosis and resulted in lower mortality compared to wildtype mice [18]. In a rat model, differential Rac2 methylation was found in animals with acute lung injury induced by lipopolysaccharide compared to controls [19]. Although bleomycin-induced pneumonitis was not the endpoint of the current GWAS, it is a well-known side-effect of bleomycin in TC patients that originates from endothelial activation and is linked to endothelial dysfunction [20, 21]. Indeed, RAC2 has been implicated in endothelial activation and neovascularization, as well as leukocyte adhesion to the endothelial cell [16, 22]. In an atherosclerosis model, Rac2 prevented plaque calcification by suppressing macrophage IL-1β expression. Furthermore, decreased RAC2 expression and increased IL-1β expression were found in calcified coronary arteries from patients [23]. Rac1/2 pathways were involved in vascular injury in diabetic mice [24]. RAC3 has been suggested to inhibit senescence, and RAC3 expression is involved in the inflammatory response after TNF stimulation [25, 26]. Moreover, Rac3 modifies the induction of endothelial dysfunction by oxidized low-density lipoprotein in human umbilical vein endothelial cells [17]. Thus, the RAC2/RAC3 network may play a role in atherosclerosis and senescence—pathophysiologic processes that have been implicated in the progress of CVD in TC patients—as well as bleomycin-induced pneumonitis. Interestingly, SNPs in RAC2 have also been found associated to cardiotoxicity due to anthracycline chemotherapy, suggesting that the RAC2/RAC3 network is a common denominator in cardiovascular toxicity of multiple chemotherapeutic agents [27]. The occurrence of pathophysiological processes in which the RAC2/RAC3 network is involved, is illustrated in a cohort of 60 TC patients with the observation that the number of CECs increases during consecutive cycles of platinum-based chemotherapy as sign of endothelial activation.

The biological themes of metabolism and adiposity, immune response, and caspase cascade/apoptosis are of particular interest, because these processes have been linked in literature to cardiovascular toxicity of platinum chemotherapy. The role of metabolism, adiposity, and endocrine dysfunction in the development of CVD has been well described in TC patients [28–30]. Besides this, the role of inflammation and immune response has been established in murine and cell models of platinum-induced nephrotoxicity, evidenced by upregulation of TNF-α and a direct mediating role of T lymphocytes [31–35]. The biological theme involving caspase activation and apoptosis is illustrated by murine and in vitro experiments indicating the role of caspase 1 and caspase 3 in platinum-induced nephrotoxicity, as well as endothelial cell apoptosis in response to cisplatin administration [20, 36, 37].

The low absolute incidence of TC and of CVD in chemotherapy-treated TC patients poses a challenge to perform meaningful GWAS on toxicity. Therefore, the current exploratory GWAS aimed to find relevant gene sets and possible biological themes rather than specific SNPs. To this end, the study was designed to minimize type II statistical errors whilst accepting a higher probability of type I statistical errors: in the trade-off between false positives and negatives, we avoided false negatives. Consequently, the current study should be regarded as hypothesis-generating and the highlighted biological themes should be regarded as providing a promising base for future studies on genetic susceptibility and relevant biomarkers in CVD in TC patients. In this regard, results from the ongoing trials on genetic variation in TC survivors in relation to renal and cardiovascular toxicity (NCT02303015), as well as ototoxicity and neurotoxicity (NCT02890030, NCT02677727) are awaited.

The need to study SNPs associated with CVD in the specific population of TC patients derives from the notion that involved SNPs might differ from the currently known SNPs associated with increased risk of CVD in the general population. Indeed, only two of the 179 SNPs associated with CVD in the current GWAS were recorded to be associated with any cardiovascular phenotype in the NHGRI-EBI Catalog of published GWAS (catalog release 11 December 2017): rs2130392 and rs6773957 were associated with Kawasaki syndrome and adiponectin levels, respectively [38]. Nevertheless, future research should not only aim at exploring and validating results from genetic studies in TC cohorts, such as the biological themes highlighted in the current analysis, but also investigate the value of genetic risk scores derived from CVD GWAS in the general population.

The major strengths of this GWAS are the well-defined cohort, the completeness of follow-up, and the clinical relevance of addressing susceptibility to CVD in TC patients. Two additional remarks should be made on the study design. First, a broad definition of CVD (both venous and arterial) was used to define cases, because a stricter definition of only arterial events would unwarrantably compromise statistical power. Second, as we included patients treated from 1977 to 2011, follow-up duration varied widely, although selection bias in this regard may be considered unlikely given only a 1 year longer median follow-up duration for the cases compared to the control group, and equal ranges of follow-up duration for both groups.

## Conclusions

In this exploratory GWAS, ten biological themes were linked with the occurrence of CVD in platinum-treated TC patients. These biological themes include metabolism and adipositas, immune response, apoptosis, and most prominently the RAC2/RAC3 network. This network has been implicated in bleomycin-induced lung injury, vascular oxidative stress, premature senescence, and endothelial activation. The biology of the RAC network was illustrated by observed CEC induction as sign of endothelial activation during consecutive courses of cisplatin-based chemotherapy in a TC cohort. Insight in the genetic variants determining susceptibility to CVD in TC patients can aid in the development of intervention strategies to prevent long-term sequelae of chemotherapy in often young cancer survivors.

## Supplementary information

Supplementary material
