# Supplementary material for: Genome-wide association study of cardiovascular disease in testicular cancer patients treated with platinum-based chemotherapy
Source: Pharmacogenomics J. 2020 Oct 3;21(2):152–64. doi: 10.1038/s41397-020-00191-8 (PMC7997802; doi:10.1038/s41397-020-00191-8)
Supplement: Supplementary file 1 — Supplementary material [file 41397_2020_191_MOESM1_ESM.pdf]

Supplementary Table S1. Overview of the 179 SNPs that were associated at p ≤ 0.001 with the occurrence of any cardiovascular event during or after chemotherapy. Reference SNP cluster IDs are reported for each SNP. P is the empirical P for association using the permutation procedure. SNP: single nucleotide polymorphism.

**SNP**	***p***
rs12692720	4,00E-05
rs1352436	4,00E-05
rs983098	4,00E-05
rs199635	4,00E-05
rs34814294	4,00E-05
rs3849324	8,00E-05
rs4755718	8,00E-05
rs7939586	8,00E-05
rs6538046	8,00E-05
rs1025206	8,00E-05
rs16970618	8,00E-05
rs11874286	8,00E-05
rs6687976	0,00012
rs7744306	0,00012
rs10950657	0,00012
rs2123269	0,00012
rs10768008	0,00012
rs10932020	0,00016
rs4466027	0,00016
rs9459964	0,00016
rs6988639	0,00016
rs12439991	0,00016
rs2331545	0,0002
rs6813846	0,0002
rs10461655	0,0002
rs7748814	0,0002
rs676740	0,0002
rs12273774	0,0002
rs4755689	0,0002
rs11164896	0,00024
rs3934720	0,00024
rs13121254	0,00024
rs10034996	0,00024
rs755535	0,00024
rs7702793	0,00024
rs9324446	0,00024
rs1826613	0,00024
rs1263635	0,00024
rs943888	0,00024
rs1387092	0,00028
rs4756786	0,00028
rs4757245	0,00028
rs11582429	0,00032
rs4858795	0,00032

rs6816525	0,00032
rs4896501	0,00032
rs17377955	0,00032
rs2554728	0,00032
rs10503759	0,00032
rs10828065	0,00032
rs17790008	0,00032
rs596557	0,00032
rs9949956	0,00032
rs4662553	0,00036
rs7745485	0,00036
rs17756443	0,00036
rs12920637	0,00036
rs5931289	0,00036
rs2215375	0,0004
rs4432837	0,0004
rs11772261	0,0004
rs13243936	0,0004
rs6467607	0,0004
rs7101204	0,0004
rs7929359	0,0004
rs10902531	0,0004
rs6692	0,0004
rs1555145	0,0004
rs4676617	0,00044
rs11736162	0,00044
rs2130392	0,00044
rs9459963	0,00044
rs2040664	0,00044
rs6462780	0,00044
rs11025878	0,00044
rs11066610	0,00044
rs11066638	0,00044
rs2027469	0,00048
rs11800877	0,00048
rs2275696	0,00048
rs3866223	0,00048
rs1366906	0,00048
rs12688573	0,00048
rs12198618	0,00052
rs4237648	0,00052
rs11643432	0,00052
rs28890299	0,00052
rs2070584	0,00052
rs10182928	0,00056
rs7610664	0,00056
rs4689203	0,00056
rs239953	0,00056
rs6558831	0,00056
rs10822863	0,00056

rs2894011	0,00056
rs4751878	0,00056
rs9530423	0,00056
rs7143719	0,00056
rs12965155	0,00056
rs760150	0,00056
rs2341921	0,00056
rs758439	0,00056
rs617459	0,0006
rs12165104	0,0006
rs11688528	0,00064
rs6759648	0,00064
rs2973419	0,00064
rs10456118	0,00064
rs6966799	0,00064
rs4644637	0,00064
rs4795934	0,00064
rs2168951	0,00068
rs1375547	0,00068
rs9880546	0,00068
rs16999330	0,00068
rs6557678	0,00068
rs7831168	0,00068
rs16993897	0,00068
rs4528743	0,00072
rs13121492	0,00072
rs40566	0,00072
rs4947522	0,00072
rs13249135	0,00072
rs16880318	0,00072
rs747925	0,00072
rs16942882	0,00072
rs17078840	0,00072
rs12457667	0,00072
rs3911618	0,00076
rs9822731	0,00076
rs7691972	0,00076
rs7722584	0,00076
rs3812278	0,00076
rs10505371	0,00076
rs6507498	0,00076
rs6673313	0,0008
rs6773957	0,0008
rs4762060	0,0008
rs10858680	0,0008
rs11610234	0,0008
rs8030490	0,0008
rs4577099	0,0008
rs1415439	0,00084
rs7593846	0,00084

rs2147866	0,00084
rs4607409	0,00084
rs4723679	0,00084
rs2506145	0,00084
rs898918	0,00084
rs12100703	0,00084
rs13406850	0,00088
rs7837472	0,00088
rs8178838	0,00088
rs6432774	0,00092
rs7673254	0,00092
rs949719	0,00092
rs745247	0,00092
rs3003177	0,00092
rs6934819	0,00092
rs11136689	0,00092
rs10764344	0,00092
rs7916162	0,00092
rs9555784	0,00092
rs2236570	0,00096
rs4521178	0,00096
rs9864293	0,00096
rs938025	0,00096
rs2201369	0,00096
rs10868152	0,00096
rs1400438	0,00096
rs11640395	0,00096
rs8095771	0,00096
rs12750904	0,001
rs2100346	0,001
rs907991	0,001
rs4143863	0,001
rs10501827	0,001
rs8069972	0,001
rs8064765	0,001

Supplementary Table S2. DEPICT reported 33 enriched gene sets. ENSG: Ensembl gene; GO: gene ontology; HGNC: Human Genome Organisation Gene Nomenclature Committee; KEGG: Kyoto Encyclopedia of Genes and Genomes; MP: mammalian phenotype ontology.

#	Gene set description (DEPICT gene set source)	Gene set ID provided by DEPICT	Nominal *p*	log(nominal *p*)
1	RAC3 subnetwork (ENSG)	ENSG00000169750	$1.18 \cdot 10^{-05}$	-4,928117993
2	RAC2 subnetwork (ENSG)	ENSG00000128340	$1.25 \cdot 10^{-05}$	-4,903089987
3	Non-small cell lung cancer (KEGG)	KEGG_NON_SMALL_CELL_LUNG_CANCER	$3.19 \cdot 10^{-05}$	-4,496209317
4	RAF1 subnetwork (ENSG)	ENSG00000132155	$5.23 \cdot 10^{-05}$	-4,281498311
5	NAT9 subnetwork (ENSG)	ENSG00000109065	$1.10 \cdot 10^{-04}$	-3,958607315
6	Acute myeloid leukemia (KEGG)	KEGG_ACUTE_MYELOID_LEUKEMIA	$1.40 \cdot 10^{-04}$	-3,853871964
7	Protein serine/threonine/tyrosine kinase activity (GO)	GO:0004712	$1.63 \cdot 10^{-04}$	-3,787812396
8	CTNNB1 subnetwork (ENSG)	ENSG00000168036	$1.78 \cdot 10^{-04}$	-3,749579998
9	Abnormal osteoclast differentiation (MP)	MP:0008396	$1.90 \cdot 10^{-04}$	-3,721246399
10	CEBPB subnetwork (ENSG)	ENSG00000172216	$2.23 \cdot 10^{-04}$	-3,651695137
11	MAP kinase kinase activity (GO)	GO:0004708	$2.83 \cdot 10^{-04}$	-3,548213564
12	Signalling by NGF (Reactome)	REACTOME_SIGNALLING_BY_NGF	$2.98 \cdot 10^{-04}$	-3,525783736
13	Increased percent body fat (MP)	MP:0005458	$3.31 \cdot 10^{-04}$	-3,480172006
14	Prolonged estrous cycle (MP)	MP:0009006	$3.54 \cdot 10^{-04}$	-3,450996738
15	Downstream TCR signaling (Reactome)	REACTOME_DOWNSTREAM_TCR_SIGNALING	$3.81 \cdot 10^{-04}$	-3,419075024
16	Downstream signal transduction (Reactome)	REACTOME_DOWNSTREAM_SIGNAL_TRANSDUCTION	$4.06 \cdot 10^{-04}$	-3,391473966
17	DAG and IP3 signaling (Reactome)	REACTOME_DAG_AND_IP3_SIGNALING	$4.18 \cdot 10^{-04}$	-3,378823718
18	Phosphoric ester hydrolase activity (GO)	GO:0042578	$4.19 \cdot 10^{-04}$	-3,377785977
19	TGOLN2 subnetwork (ENSG)	ENSG00000152291	$4.96 \cdot 10^{-04}$	-3,304518324
20	EGFR interacts with PLCG1 (Reactome)	REACTOME_EGFR_INTERACTS_WITH_PHOSPHOLIPASE_C:GAMMA	$5.22 \cdot 10^{-04}$	-3,282329497
21	TCR signaling (Reactome)	REACTOME_TCR_SIGNALING	$5.33 \cdot 10^{-04}$	-3,273272791
22	PLCG1 events in ERBB2 signaling (Reactome)	REACTOME_PLCG1_EVENTS_IN_ERBB2_SIGNALING	$5.68 \cdot 10^{-04}$	-3,245651664
23	MAP2K1 subnetwork (ENSG)	ENSG00000169032	$7.12 \cdot 10^{-04}$	-3,147520006
24	NGF signalling via TRKA from the plasma membrane (Reactome)	REACTOME_NGF_SIGNALLING_VIA_TRKA_FROM_THE_PLASMA_MEMBRANE	$7.97 \cdot 10^{-04}$	-3,098541679
25	Enlarged lymph nodes (MP)	MP:0000702	$8.06 \cdot 10^{-04}$	-3,093664958
26	NOD1 subnetwork (ENSG)	ENSG00000106100	$8.08 \cdot 10^{-04}$	-3,092588639
27	Activation of immune response (GO)	GO:0002253	$8.14 \cdot 10^{-04}$	-3,089375595
28	Phosphoprotein phosphatase activity (GO)	GO:0004721	$8.15 \cdot 10^{-04}$	-3,088842391
29	NGF receptor signaling pathway (GO)	GO:0048011	$8.19 \cdot 10^{-04}$	-3,086716098
30	Phosphatase activity (GO)	GO:0016791	$8.64 \cdot 10^{-04}$	-3,063486258
31	Reduced female fertility (MP)	MP:0001923	$8.98 \cdot 10^{-04}$	-3,046723663
32	Protein kinase binding (GO)	GO:0019901	$9.31 \cdot 10^{-04}$	-3,031050319
33	Kinase binding (GO)	GO:0019900	$9.54 \cdot 10^{-04}$	-3,020451625

Supplementary Table S3. Representative genes for the biological themes. To understand the biological themes, we determined the top hundred representative genes for each gene set cluster. These genes had the highest absolute weighted mean z-score in the gene set cluster, using the multiplicative inverse of the nominal P of each reconstituted gene set as a weight for each z-score. ENSG: Ensembl gene; GO: gene ontology; KEGG: Kyoto Encyclopedia of Genes and Genomes; MP: mammalian phenotype ontology.

**Biological theme:**	**RAC2/RAC3 network**
**Gene sets:**	RAC3 subnetwork (ENSG)
	RAC2 subnetwork (ENSG)

Ensembl gene ID	Gene	Weighted mean *z* -score	Description
ENSG00000114331	ACAP2	4,082266	ArfGAP with coiled-coil, ankyrin repeat and PH domains 2 [Source:HGNC Symbol;Acc:HGNC:16469]
ENSG00000136167	LCP1	3,869325	lymphocyte cytosolic protein 1 [Source:HGNC Symbol;Acc:HGNC:6528]
ENSG00000108175	ZMIZ1	3,788778	zinc finger MIZ-type containing 1 [Source:HGNC Symbol;Acc:HGNC:16493]
ENSG00000117676	RPS6KA1	3,718398	ribosomal protein S6 kinase A1 [Source:HGNC Symbol;Acc:HGNC:10430]
ENSG00000134982	APC	3,685608	APC, WNT signaling pathway regulator [Source:HGNC Symbol;Acc:HGNC:583]
ENSG00000167377	ZNF23	-3,57116	zinc finger protein 23 [Source:HGNC Symbol;Acc:HGNC:13023]
ENSG00000129437	KLK14	-3,54709	kallikrein related peptidase 14 [Source:HGNC Symbol;Acc:HGNC:6362]
ENSG00000158985	CDC42SE2	3,52261	CDC42 small effector 2 [Source:HGNC Symbol;Acc:HGNC:18547]
ENSG00000132334	PTPRE	3,519643	protein tyrosine phosphatase, receptor type E [Source:HGNC Symbol;Acc:HGNC:9669]
ENSG00000198879	SFMBT2	3,47089	Scm-like with four mbt domains 2 [Source:HGNC Symbol;Acc:HGNC:20256]
ENSG00000184640	SEPT9	3,456023	septin 9 [Source:HGNC Symbol;Acc:HGNC:7323]
ENSG00000107099	DOCK8	3,406204	dedicator of cytokinesis 8 [Source:HGNC Symbol;Acc:HGNC:19191]
ENSG00000089163	SIRT4	-3,3535	sirtuin 4 [Source:HGNC Symbol;Acc:HGNC:14932]
ENSG00000142669	SH3BGRL3	3,35287	SH3 domain binding glutamate rich protein like 3 [Source:HGNC Symbol;Acc:HGNC:15568]
ENSG00000186517	ARHGAP30	3,33409	Rho GTPase activating protein 30 [Source:HGNC Symbol;Acc:HGNC:27414]
ENSG00000134516	DOCK2	3,32512	dedicator of cytokinesis 2 [Source:HGNC Symbol;Acc:HGNC:2988]
ENSG00000165813	CCDC186	3,31568	coiled-coil domain containing 186 [Source:HGNC Symbol;Acc:HGNC:24349]
ENSG00000115756	HPCAL1	3,306617	hippocalcin like 1 [Source:HGNC Symbol;Acc:HGNC:5145]
ENSG00000230937	MIR205HG	3,278021	MIR205 host gene [Source:HGNC Symbol;Acc:HGNC:43562]
ENSG00000105388	CEACAM5	-3,26759	carcinoembryonic antigen related cell adhesion molecule 5 [Source:HGNC Symbol;Acc:HGNC:1817]
ENSG00000179195	ZNF664	-3,26443	zinc finger protein 664 [Source:HGNC Symbol;Acc:HGNC:25406]
ENSG00000068724	TTC7A	3,259161	tetratricopeptide repeat domain 7A [Source:HGNC Symbol;Acc:HGNC:19750]
ENSG00000107263	RAPGEF1	3,251132	Rap guanine nucleotide exchange factor 1 [Source:HGNC Symbol;Acc:HGNC:4568]
ENSG00000160271	RALGDS	3,24888	ral guanine nucleotide dissociation stimulator [Source:HGNC Symbol;Acc:HGNC:9842]
ENSG00000159840	ZYX	3,244708	zyxin [Source:HGNC Symbol;Acc:HGNC:13200]
ENSG00000142634	EFHD2	3,20519	EF-hand domain family member D2 [Source:HGNC Symbol;Acc:HGNC:28670]
ENSG00000155629	PIK3AP1	3,195289	phosphoinositide-3-kinase adaptor protein 1 [Source:HGNC Symbol;Acc:HGNC:30034]
ENSG00000129625	REEP5	3,189271	receptor accessory protein 5 [Source:HGNC Symbol;Acc:HGNC:30077]

ENSG00000131236	CAP1	3,132347	adenylate cyclase associated protein 1 [Source:HGNC Symbol;Acc:HGNC:20040]
ENSG00000162704	ARPC5	3,130825	actin related protein 2/3 complex subunit 5 [Source:HGNC Symbol;Acc:HGNC:708]
ENSG00000143669	LYST	3,102473	lysosomal trafficking regulator [Source:HGNC Symbol;Acc:HGNC:1968]
ENSG00000114316	USP4	3,100548	ubiquitin specific peptidase 4 [Source:HGNC Symbol;Acc:HGNC:12627]
ENSG00000100263	RHBDD3	-3,09891	rhomboid domain containing 3 [Source:HGNC Symbol;Acc:HGNC:1308]
ENSG00000198561	CTNND1	3,088787	catenin delta 1 [Source:HGNC Symbol;Acc:HGNC:2515]
ENSG00000144228	SPOPL	3,083418	speckle type BTB/POZ protein like [Source:HGNC Symbol;Acc:HGNC:27934]
ENSG00000177764	NA	-3,08322	NA
ENSG00000066084	DIP2B	3,0802	disco interacting protein 2 homolog B [Source:HGNC Symbol;Acc:HGNC:29284]
ENSG00000235687	LINC00993	3,079696	long intergenic non-protein coding RNA 993 [Source:HGNC Symbol;Acc:HGNC:48948]
ENSG00000186806	VSIG10L	-3,06363	V-set and immunoglobulin domain containing 10 like [Source:HGNC Symbol;Acc:HGNC:27111]
ENSG00000100345	MYH9	3,062658	myosin heavy chain 9 [Source:HGNC Symbol;Acc:HGNC:7579]
ENSG00000197622	CDC42SE1	3,050791	CDC42 small effector 1 [Source:HGNC Symbol;Acc:HGNC:17719]
ENSG00000008196	TFAP2B	3,049955	transcription factor AP-2 beta [Source:HGNC Symbol;Acc:HGNC:11743]
ENSG00000140455	USP3	3,048126	ubiquitin specific peptidase 3 [Source:HGNC Symbol;Acc:HGNC:12626]
ENSG00000159899	NPR2	-3,04151	natriuretic peptide receptor 2 [Source:HGNC Symbol;Acc:HGNC:7944]
ENSG00000139668	WDFY2	3,038018	WD repeat and FYVE domain containing 2 [Source:HGNC Symbol;Acc:HGNC:20482]
ENSG00000050030	KIAA2022	3,035292	KIAA2022 [Source:HGNC Symbol;Acc:HGNC:29433]
ENSG00000161202	DVL3	3,020795	dishevelled segment polarity protein 3 [Source:HGNC Symbol;Acc:HGNC:3087]
ENSG00000138071	ACTR2	3,016839	ARP2 actin related protein 2 homolog [Source:HGNC Symbol;Acc:HGNC:169]
ENSG00000175376	EIF1AD	-3,00443	eukaryotic translation initiation factor 1A domain containing [Source:HGNC Symbol;Acc:HGNC:28147]
ENSG00000248762		-3,0028	
ENSG00000163466	ARPC2	2,987742	actin related protein 2/3 complex subunit 2 [Source:HGNC Symbol;Acc:HGNC:705]
ENSG00000180353	HCLS1	2,981743	hematopoietic cell-specific Lyn substrate 1 [Source:HGNC Symbol;Acc:HGNC:4844]
ENSG00000185862	EVI2B	2,976875	ecotropic viral integration site 2B [Source:HGNC Symbol;Acc:HGNC:3500]
ENSG00000130755	GMFG	2,967221	glia maturation factor gamma [Source:HGNC Symbol;Acc:HGNC:4374]
ENSG00000089639	GMIP	2,965812	GEM interacting protein [Source:HGNC Symbol;Acc:HGNC:24852]
ENSG00000129167	TPH1	-2,96509	tryptophan hydroxylase 1 [Source:HGNC Symbol;Acc:HGNC:12008]
ENSG00000101265	RASSF2	2,964265	Ras association domain family member 2 [Source:HGNC Symbol;Acc:HGNC:9883]
ENSG00000143119	CD53	2,963863	CD53 molecule [Source:HGNC Symbol;Acc:HGNC:1686]
ENSG00000078369	GNB1	2,962238	G protein subunit beta 1 [Source:HGNC Symbol;Acc:HGNC:4396]
ENSG00000137198	GMPR	-2,95042	guanosine monophosphate reductase [Source:HGNC Symbol;Acc:HGNC:4376]
ENSG00000087253	LPCAT2	2,949326	lysophosphatidylcholine acyltransferase 2 [Source:HGNC Symbol;Acc:HGNC:26032]
ENSG00000167613	LAIR1	2,946114	leukocyte associated immunoglobulin like receptor 1 [Source:HGNC Symbol;Acc:HGNC:6477]
ENSG00000166912	MTMR10	2,941512	myotubularin related protein 10 [Source:HGNC Symbol;Acc:HGNC:25999]
ENSG00000109062	SLC9A3R1	2,939624	SLC9A3 regulator 1 [Source:HGNC Symbol;Acc:HGNC:11075]
ENSG00000133678	TMEM254	2,934218	transmembrane protein 254 [Source:HGNC Symbol;Acc:HGNC:25804]

ENSG00000140368	PSTPIP1	2,920872	proline-serine-threonine phosphatase interacting protein 1 [Source:HGNC Symbol;Acc:HGNC:9580]
ENSG00000143850	PLEKHA6	2,911262	pleckstrin homology domain containing A6 [Source:HGNC Symbol;Acc:HGNC:17053]
ENSG00000230068	CDC42-IT1	2,909927	CDC42 intronic transcript 1 [Source:HGNC Symbol;Acc:HGNC:41317]
ENSG00000162511	LAPTM5	2,906254	lysosomal protein transmembrane 5 [Source:HGNC Symbol;Acc:HGNC:29612]
ENSG00000072786	STK10	2,904434	serine/threonine kinase 10 [Source:HGNC Symbol;Acc:HGNC:11388]
ENSG00000254936		-2,89964	
ENSG00000077549	CAPZB	2,898779	capping actin protein of muscle Z-line beta subunit [Source:HGNC Symbol;Acc:HGNC:1491]
ENSG00000115091	ACTR3	2,895413	ARP3 actin related protein 3 homolog [Source:HGNC Symbol;Acc:HGNC:170]
ENSG00000162571	TTLL10	2,893898	tubulin tyrosine ligase like 10 [Source:HGNC Symbol;Acc:HGNC:26693]
ENSG00000101132	PFDN4	-2,88356	prefoldin subunit 4 [Source:HGNC Symbol;Acc:HGNC:8868]
ENSG00000232267	ACTR3P2	2,883317	ACTR3 pseudogene 2 [Source:HGNC Symbol;Acc:HGNC:38676]
ENSG00000125089	SH3TC1	2,881901	SH3 domain and tetratricopeptide repeats 1 [Source:HGNC Symbol;Acc:HGNC:26009]
ENSG00000141480	ARRB2	2,881584	arrestin beta 2 [Source:HGNC Symbol;Acc:HGNC:712]
ENSG00000172795	DCP2	2,880332	decapping mRNA 2 [Source:HGNC Symbol;Acc:HGNC:24452]
ENSG00000148396	SEC16A	2,877623	SEC16 homolog A, endoplasmic reticulum export factor [Source:HGNC Symbol;Acc:HGNC:29006]
ENSG00000138382	METTL5	-2,87192	methyltransferase like 5 [Source:HGNC Symbol;Acc:HGNC:25006]
ENSG00000101336	HCK	2,865032	HCK proto-oncogene, Src family tyrosine kinase [Source:HGNC Symbol;Acc:HGNC:4840]
ENSG00000188153	COL4A5	2,848853	collagen type IV alpha 5 chain [Source:HGNC Symbol;Acc:HGNC:2207]
ENSG00000126860	EVI2A	2,846796	ecotropic viral integration site 2A [Source:HGNC Symbol;Acc:HGNC:3499]
ENSG00000186526	CYP4F8	2,8426	cytochrome P450 family 4 subfamily F member 8 [Source:HGNC Symbol;Acc:HGNC:2648]
ENSG00000162373	BEND5	2,840534	BEN domain containing 5 [Source:HGNC Symbol;Acc:HGNC:25668]
ENSG00000111404	RERGL	2,838674	RERG like [Source:HGNC Symbol;Acc:HGNC:26213]
ENSG00000131480	AOC2	-2,83776	amine oxidase, copper containing 2 [Source:HGNC Symbol;Acc:HGNC:549]
ENSG00000236268	LINC01361	-2,83764	long intergenic non-protein coding RNA 1361 [Source:HGNC Symbol;Acc:HGNC:50595]
ENSG00000153823	PID1	2,835276	phosphotyrosine interaction domain containing 1 [Source:HGNC Symbol;Acc:HGNC:26084]
ENSG00000083307	GRHL2	2,831656	grainyhead like transcription factor 2 [Source:HGNC Symbol;Acc:HGNC:2799]
ENSG00000113231	PDE8B	2,820239	phosphodiesterase 8B [Source:HGNC Symbol;Acc:HGNC:8794]
ENSG00000100842	EFS	2,818926	embryonal Fyn-associated substrate [Source:HGNC Symbol;Acc:HGNC:16898]
ENSG00000137486	ARRB1	2,811192	arrestin beta 1 [Source:HGNC Symbol;Acc:HGNC:711]
ENSG00000120162	MOB3B	2,809979	MOB kinase activator 3B [Source:HGNC Symbol;Acc:HGNC:23825]
ENSG00000172183	ISG20	-2,80719	interferon stimulated exonuclease gene 20 [Source:HGNC Symbol;Acc:HGNC:6130]
ENSG00000072518	MARK2	2,804749	microtubule affinity regulating kinase 2 [Source:HGNC Symbol;Acc:HGNC:3332]
ENSG00000137642	SORL1	2,802471	sortilin related receptor 1 [Source:HGNC Symbol;Acc:HGNC:11185]
ENSG00000125741	OPA3	-2,79723	OPA3, outer mitochondrial membrane lipid metabolism regulator [Source:HGNC Symbol;Acc:HGNC:8142]
ENSG00000204856	FAM216A	-2,79366	family with sequence similarity 216 member A [Source:HGNC Symbol;Acc:HGNC:30180]

**Biological theme:**	**NAT9 network**
**Gene sets:**	NAT9 subnetwork (ENSG)

**Ensembl gene ID**	**Gene**	**Weighted mean *z* -score**	**Description**
ENSG00000133606	MKRN1	5,163726	makorin ring finger protein 1 [Source:HGNC Symbol;Acc:HGNC:7112]
ENSG00000072042	RDH11	4,870137	retinol dehydrogenase 11 (all-trans/9-cis/11-cis) [Source:HGNC Symbol;Acc:HGNC:17964]
ENSG00000237940	LINC01238	4,48427	long intergenic non-protein coding RNA 1238 [Source:HGNC Symbol;Acc:HGNC:49795]
ENSG00000213047	DENND1B	-4,40425	DENN domain containing 1B [Source:HGNC Symbol;Acc:HGNC:28404]
ENSG00000132604	TERF2	4,313336	telomeric repeat binding factor 2 [Source:HGNC Symbol;Acc:HGNC:11729]
ENSG00000113552	GNPDA1	4,02803	glucosamine-6-phosphate deaminase 1 [Source:HGNC Symbol;Acc:HGNC:4417]
ENSG00000197930	ERO1A	3,940358	endoplasmic reticulum oxidoreductase 1 alpha [Source:HGNC Symbol;Acc:HGNC:13280]
ENSG00000183166	CALN1	3,931943	calneuron 1 [Source:HGNC Symbol;Acc:HGNC:13248]
ENSG00000227051	C14orf132	3,900367	chromosome 14 open reading frame 132 [Source:HGNC Symbol;Acc:HGNC:20346]
ENSG00000173681	CXorf23	-3,8711	chromosome X open reading frame 23 [Source:HGNC Symbol;Acc:HGNC:27413]
ENSG00000213240		-3,87008	
ENSG00000139625	MAP3K12	3,849265	mitogen-activated protein kinase kinase kinase 12 [Source:HGNC Symbol;Acc:HGNC:6851]
ENSG00000196123	KIAA0895L	3,786199	KIAA0895 like [Source:HGNC Symbol;Acc:HGNC:34408]
ENSG00000127325	BEST3	3,738082	bestrophin 3 [Source:HGNC Symbol;Acc:HGNC:17105]
ENSG00000151612	ZNF827	3,715385	zinc finger protein 827 [Source:HGNC Symbol;Acc:HGNC:27193]
ENSG00000157741	UBN2	3,692975	ubinuclein 2 [Source:HGNC Symbol;Acc:HGNC:21931]
ENSG00000225986	UBXN10-AS1	-3,63966	UBXN10 antisense RNA 1 [Source:HGNC Symbol;Acc:HGNC:41141]
ENSG00000005483	KMT2E	3,600568	lysine methyltransferase 2E [Source:HGNC Symbol;Acc:HGNC:18541]
ENSG00000166848	TERF2IP	3,588258	TERF2 interacting protein [Source:HGNC Symbol;Acc:HGNC:19246]
ENSG00000101746	NOL4	3,569744	nucleolar protein 4 [Source:HGNC Symbol;Acc:HGNC:7870]
ENSG00000127995	CASD1	3,514453	CAS1 domain containing 1 [Source:HGNC Symbol;Acc:HGNC:16014]
ENSG00000008083	JARID2	-3,51418	jumonji and AT-rich interaction domain containing 2 [Source:HGNC Symbol;Acc:HGNC:6196]
ENSG00000139410	SDSL	-3,50504	serine dehydratase like [Source:HGNC Symbol;Acc:HGNC:30404]
ENSG00000196968	FUT11	3,472206	fucosyltransferase 11 [Source:HGNC Symbol;Acc:HGNC:19233]
ENSG00000163516	ANKZF1	3,471064	ankyrin repeat and zinc finger domain containing 1 [Source:HGNC Symbol;Acc:HGNC:25527]
ENSG00000225377	NRSN2-AS1	3,470431	NRSN2 antisense RNA 1 [Source:HGNC Symbol;Acc:HGNC:51222]
ENSG00000071553	ATP6AP1	3,429073	ATPase H+ transporting accessory protein 1 [Source:HGNC Symbol;Acc:HGNC:868]
ENSG00000197826	C4orf22	3,423673	chromosome 4 open reading frame 22 [Source:HGNC Symbol;Acc:HGNC:28554]
ENSG00000083457	ITGAE	-3,38662	integrin subunit alpha E [Source:HGNC Symbol;Acc:HGNC:6147]
ENSG00000167508	MVD	3,376736	mevalonate diphosphate decarboxylase [Source:HGNC Symbol;Acc:HGNC:7529]
ENSG00000176049	JAKMIP2	3,376575	janus kinase and microtubule interacting protein 2 [Source:HGNC Symbol;Acc:HGNC:29067]
ENSG00000185928	NA	3,358964	NA
ENSG00000168824		3,341393	Neuron-specific protein family member 1 [Source:UniProtKB/Swiss-Prot;Acc:P42857]

ENSG00000122299	ZC3H7A	3,321419	zinc finger CCCH-type containing 7A [Source:HGNC Symbol;Acc:HGNC:30959]
ENSG00000166963	MAP1A	3,286456	microtubule associated protein 1A [Source:HGNC Symbol;Acc:HGNC:6835]
ENSG00000250582	SMAD1-AS2	3,279362	SMAD1 antisense RNA 2 [Source:HGNC Symbol;Acc:HGNC:49381]
ENSG00000196440	ARMCX4	3,260547	armadillo repeat containing, X-linked 4 [Source:HGNC Symbol;Acc:HGNC:28615]
ENSG00000119669	IRF2BPL	3,256765	interferon regulatory factor 2 binding protein like [Source:HGNC Symbol;Acc:HGNC:14282]
ENSG00000254815		3,247514	
ENSG00000245008		3,242292	
ENSG00000152208	GRID2	3,238902	glutamate ionotropic receptor delta type subunit 2 [Source:HGNC Symbol;Acc:HGNC:4576]
ENSG00000105438	KDELR1	3,224069	KDEL endoplasmic reticulum protein retention receptor 1 [Source:HGNC Symbol;Acc:HGNC:6304]
ENSG00000141279	NPEPPS	3,21015	aminopeptidase puromycin sensitive [Source:HGNC Symbol;Acc:HGNC:7900]
ENSG00000154143	PANX3	3,198579	pannexin 3 [Source:HGNC Symbol;Acc:HGNC:20573]
ENSG00000180773	SLC36A4	3,18908	solute carrier family 36 member 4 [Source:HGNC Symbol;Acc:HGNC:19660]
ENSG00000135502	SLC26A10	3,17146	solute carrier family 26 member 10 [Source:HGNC Symbol;Acc:HGNC:14470]
ENSG00000109046	WSB1	3,16954	WD repeat and SOCS box containing 1 [Source:HGNC Symbol;Acc:HGNC:19221]
ENSG00000151575	TEX9	3,164131	testis expressed 9 [Source:HGNC Symbol;Acc:HGNC:29585]
ENSG00000198105	ZNF248	3,162619	zinc finger protein 248 [Source:HGNC Symbol;Acc:HGNC:13041]
ENSG00000184939	ZFP90	3,146268	ZFP90 zinc finger protein [Source:HGNC Symbol;Acc:HGNC:23329]
ENSG00000172766	NAA16	3,140273	N(alpha)-acetyltransferase 16, NatA auxiliary subunit [Source:HGNC Symbol;Acc:HGNC:26164]
ENSG00000229191		-3,14024	
ENSG00000181378	CFAP65	3,139302	cilia and flagella associated protein 65 [Source:HGNC Symbol;Acc:HGNC:25325]
ENSG00000172458	IL17D	3,137064	interleukin 17D [Source:HGNC Symbol;Acc:HGNC:5984]
ENSG00000250510	GPR162	3,125326	G protein-coupled receptor 162 [Source:HGNC Symbol;Acc:HGNC:16693]
ENSG00000164038	SLC9B2	3,118095	solute carrier family 9 member B2 [Source:HGNC Symbol;Acc:HGNC:25143]
ENSG00000099194	SCD	3,099114	stearoyl-CoA desaturase [Source:HGNC Symbol;Acc:HGNC:10571]
ENSG00000114062	UBE3A	3,075079	ubiquitin protein ligase E3A [Source:HGNC Symbol;Acc:HGNC:12496]
ENSG00000178988	MRFAP1L1	3,069838	Morf4 family associated protein 1 like 1 [Source:HGNC Symbol;Acc:HGNC:28796]
ENSG00000204428	LY6G5C	3,061001	lymphocyte antigen 6 family member G5C [Source:HGNC Symbol;Acc:HGNC:13932]
ENSG00000138741	TRPC3	3,059792	transient receptor potential cation channel subfamily C member 3 [Source:HGNC Symbol;Acc:HGNC:12335]
ENSG00000007402	CACNA2D2	3,051591	calcium voltage-gated channel auxiliary subunit alpha2delta 2 [Source:HGNC Symbol;Acc:HGNC:1400]
ENSG00000107341	UBE2R2	3,051217	ubiquitin conjugating enzyme E2 R2 [Source:HGNC Symbol;Acc:HGNC:19907]
ENSG00000164211	STARD4	3,04434	StAR related lipid transfer domain containing 4 [Source:HGNC Symbol;Acc:HGNC:18058]
ENSG00000234264	DEPDC1-AS1	3,040625	DEPDC1 antisense RNA 1 [Source:HGNC Symbol;Acc:HGNC:50592]
ENSG00000237928	NFIA-AS2	3,037764	NFIA antisense RNA 2 [Source:HGNC Symbol;Acc:HGNC:40401]
ENSG00000182473	EXOC7	-3,03746	exocyst complex component 7 [Source:HGNC Symbol;Acc:HGNC:23214]
ENSG00000064999	ANKS1A	-3,03535	ankyrin repeat and sterile alpha motif domain containing 1A [Source:HGNC Symbol;Acc:HGNC:20961]
ENSG00000254506		-3,02781	
ENSG00000114738	MAPKAPK3	-3,02608	mitogen-activated protein kinase-activated protein kinase 3 [Source:HGNC Symbol;Acc:HGNC:6888]

ENSG00000145860	RNF145	3,022123	ring finger protein 145 [Source:HGNC Symbol;Acc:HGNC:20853]
ENSG00000184293	CLECL1	-3,01752	C-type lectin like 1 [Source:HGNC Symbol;Acc:HGNC:24462]
ENSG00000008300	CELSR3	3,016192	cadherin EGF LAG seven-pass G-type receptor 3 [Source:HGNC Symbol;Acc:HGNC:3230]
ENSG00000092964	DPYSL2	3,006578	dihydropyrimidinase like 2 [Source:HGNC Symbol;Acc:HGNC:3014]
ENSG00000178078	STAP2	-2,99912	signal transducing adaptor family member 2 [Source:HGNC Symbol;Acc:HGNC:30430]
ENSG00000126351	THRA	2,995614	thyroid hormone receptor, alpha [Source:HGNC Symbol;Acc:HGNC:11796]
ENSG00000136929	HEMGN	2,994556	hemogen [Source:HGNC Symbol;Acc:HGNC:17509]
ENSG00000177732	SOX12	2,993922	SRY-box 12 [Source:HGNC Symbol;Acc:HGNC:11198]
ENSG00000197457	STMN3	2,991891	stathmin 3 [Source:HGNC Symbol;Acc:HGNC:15926]
ENSG00000247095	MIR210HG	2,987062	MIR210 host gene [Source:HGNC Symbol;Acc:HGNC:39524]
ENSG00000204060	FOXO6	2,984134	forkhead box O6 [Source:HGNC Symbol;Acc:HGNC:24814]
ENSG00000132170	PPARG	-2,97811	peroxisome proliferator activated receptor gamma [Source:HGNC Symbol;Acc:HGNC:9236]
ENSG00000178802	MPI	2,97691	mannose phosphate isomerase [Source:HGNC Symbol;Acc:HGNC:7216]
ENSG00000161958	FGF11	2,976762	fibroblast growth factor 11 [Source:HGNC Symbol;Acc:HGNC:3667]
ENSG00000052795	FNIP2	2,976298	folliculin interacting protein 2 [Source:HGNC Symbol;Acc:HGNC:29280]
ENSG00000153253	SCN3A	2,971038	sodium voltage-gated channel alpha subunit 3 [Source:HGNC Symbol;Acc:HGNC:10590]
ENSG00000143367	TUFT1	-2,96776	tuftelin 1 [Source:HGNC Symbol;Acc:HGNC:12422]
ENSG00000175265	GOLGA8A	2,965306	golgin A8 family member A [Source:HGNC Symbol;Acc:HGNC:31972]
ENSG00000203867	RBM20	-2,95801	RNA binding motif protein 20 [Source:HGNC Symbol;Acc:HGNC:27424]
ENSG00000186480	INSIG1	2,94771	insulin induced gene 1 [Source:HGNC Symbol;Acc:HGNC:6083]
ENSG00000156931	VPS8	-2,93698	VPS8, CORVET complex subunit [Source:HGNC Symbol;Acc:HGNC:29122]
ENSG00000233404	NA	-2,9369	NA
ENSG00000179029	TMEM107	2,933892	transmembrane protein 107 [Source:HGNC Symbol;Acc:HGNC:28128]
ENSG00000105656	ELL	2,93334	elongation factor for RNA polymerase II [Source:HGNC Symbol;Acc:HGNC:23114]
ENSG00000144567	RETREG2	2,929924	reticulophagy regulator family member 2 [Source:HGNC Symbol;Acc:HGNC:28450]
ENSG00000088756	ARHGAP28	-2,91809	Rho GTPase activating protein 28 [Source:HGNC Symbol;Acc:HGNC:25509]
ENSG00000007968	E2F2	2,917532	E2F transcription factor 2 [Source:HGNC Symbol;Acc:HGNC:3114]
ENSG00000134815	DHX34	-2,91423	DExH-box helicase 34 [Source:HGNC Symbol;Acc:HGNC:16719]
ENSG00000185838	GNB1L	-2,90495	G protein subunit beta 1 like [Source:HGNC Symbol;Acc:HGNC:4397]
ENSG00000255559	ZNF252P-AS1	-2,90315	ZNF252P antisense RNA 1 [Source:HGNC Symbol;Acc:HGNC:27821]

**Biological theme:**	**Protein serine/threonine/tyrosine kinase activity**
**Gene sets:**	Protein serine/threonine/tyrosine kinase activity (GO)
	MAP kinase kinase activity (GO)

**Ensembl gene ID**	**Gene**	**Weighted mean *z* -score**	**Description**
ENSG00000051009	FAM160A2	3,843557	family with sequence similarity 160 member A2 [Source:HGNC Symbol;Acc:HGNC:25378]
ENSG00000177479	ARIH2	3,661596	ariadne RBR E3 ubiquitin protein ligase 2 [Source:HGNC Symbol;Acc:HGNC:690]
ENSG00000196544	BORCS6	3,547454	BLOC-1 related complex subunit 6 [Source:HGNC Symbol;Acc:HGNC:25939]
ENSG00000237512	UNC5B-AS1	-3,51724	UNC5B antisense RNA 1 [Source:HGNC Symbol;Acc:HGNC:45096]
ENSG00000179335	CLK3	3,462331	CDC like kinase 3 [Source:HGNC Symbol;Acc:HGNC:2071]
ENSG00000245719		3,432129	
ENSG00000159346	ADIPOR1	3,431398	adiponectin receptor 1 [Source:HGNC Symbol;Acc:HGNC:24040]
ENSG00000164068	RNF123	3,424813	ring finger protein 123 [Source:HGNC Symbol;Acc:HGNC:21148]
ENSG00000153250	RBMS1	3,408851	RNA binding motif single stranded interacting protein 1 [Source:HGNC Symbol;Acc:HGNC:9907]
ENSG00000091106	NLRC4	3,390213	NLR family CARD domain containing 4 [Source:HGNC Symbol;Acc:HGNC:16412]
ENSG00000093167	LRRFIP2	3,375932	LRR binding FLII interacting protein 2 [Source:HGNC Symbol;Acc:HGNC:6703]
ENSG00000070423	RNF126	3,342756	ring finger protein 126 [Source:HGNC Symbol;Acc:HGNC:21151]
ENSG00000154370	TRIM11	3,342572	tripartite motif containing 11 [Source:HGNC Symbol;Acc:HGNC:16281]
ENSG00000234336	JAZF1-AS1	3,312443	JAZF1 antisense RNA 1 [Source:HGNC Symbol;Acc:HGNC:41218]
ENSG00000204086	RPA4	3,290707	replication protein A4 [Source:HGNC Symbol;Acc:HGNC:30305]
ENSG00000171425	ZNF581	3,280113	zinc finger protein 581 [Source:HGNC Symbol;Acc:HGNC:25017]
ENSG00000006459	KDM7A	3,249877	lysine demethylase 7A [Source:HGNC Symbol;Acc:HGNC:22224]
ENSG00000065559	MAP2K4	3,24894	mitogen-activated protein kinase kinase 4 [Source:HGNC Symbol;Acc:HGNC:6844]
ENSG00000180667	YOD1	3,248244	YOD1 deubiquitinase [Source:HGNC Symbol;Acc:HGNC:25035]
ENSG00000137871	ZNF280D	3,229429	zinc finger protein 280D [Source:HGNC Symbol;Acc:HGNC:25953]
ENSG00000182500	NA	3,21945	NA
ENSG00000114268	PFKFB4	3,158344	6-phosphofructo-2-kinase/fructose-2,6-biphosphatase 4 [Source:HGNC Symbol;Acc:HGNC:8875]
ENSG00000099804	CDC34	3,117766	cell division cycle 34 [Source:HGNC Symbol;Acc:HGNC:1734]
ENSG00000172765	TMCC1	3,116088	transmembrane and coiled-coil domain family 1 [Source:HGNC Symbol;Acc:HGNC:29116]
ENSG00000089597	GANAB	-3,09935	glucosidase II alpha subunit [Source:HGNC Symbol;Acc:HGNC:4138]
ENSG00000139132	FGD4	3,090232	FYVE, RhoGEF and PH domain containing 4 [Source:HGNC Symbol;Acc:HGNC:19125]
ENSG00000181104	F2R	-3,07464	coagulation factor II thrombin receptor [Source:HGNC Symbol;Acc:HGNC:3537]
ENSG00000099204	ABLIM1	-3,06619	actin binding LIM protein 1 [Source:HGNC Symbol;Acc:HGNC:78]
ENSG00000175727	MLXIP	3,060096	MLX interacting protein [Source:HGNC Symbol;Acc:HGNC:17055]
ENSG00000128394	APOBEC3F	-3,04877	apolipoprotein B mRNA editing enzyme catalytic subunit 3F [Source:HGNC Symbol;Acc:HGNC:17356]
ENSG00000164086	DUSP7	3,021925	dual specificity phosphatase 7 [Source:HGNC Symbol;Acc:HGNC:3073]
ENSG00000204282	TNRC6C-AS1	3,008733	TNRC6C antisense RNA 1 [Source:HGNC Symbol;Acc:HGNC:44360]

ENSG00000146054	TRIM7	3,007035	tripartite motif containing 7 [Source:HGNC Symbol;Acc:HGNC:16278]
ENSG00000137764	MAP2K5	2,989935	mitogen-activated protein kinase kinase 5 [Source:HGNC Symbol;Acc:HGNC:6845]
ENSG00000214013	GANC	2,985613	glucosidase alpha, neutral C [Source:HGNC Symbol;Acc:HGNC:4139]
ENSG00000198853	RUSC2	2,966409	RUN and SH3 domain containing 2 [Source:HGNC Symbol;Acc:HGNC:23625]
ENSG00000226900		-2,96372	
ENSG00000162746	FCRLB	2,955003	Fc receptor like B [Source:HGNC Symbol;Acc:HGNC:26431]
ENSG00000118298	CA14	2,943536	carbonic anhydrase 14 [Source:HGNC Symbol;Acc:HGNC:1372]
ENSG00000233968		-2,92361	
ENSG00000146809	ASB15	2,918978	ankyrin repeat and SOCS box containing 15 [Source:HGNC Symbol;Acc:HGNC:19767]
ENSG00000144659	SLC25A38	2,908268	solute carrier family 25 member 38 [Source:HGNC Symbol;Acc:HGNC:26054]
ENSG00000179363	TMEM31	2,901561	transmembrane protein 31 [Source:HGNC Symbol;Acc:HGNC:28601]
ENSG00000085978	ATG16L1	2,88723	autophagy related 16 like 1 [Source:HGNC Symbol;Acc:HGNC:21498]
ENSG00000070759	TESK2	2,883233	testis-specific kinase 2 [Source:HGNC Symbol;Acc:HGNC:11732]
ENSG00000106991	ENG	-2,87189	endoglin [Source:HGNC Symbol;Acc:HGNC:3349]
ENSG00000093010	COMT	-2,86549	catechol-O-methyltransferase [Source:HGNC Symbol;Acc:HGNC:2228]
ENSG00000065970	FOXJ2	2,862791	forkhead box J2 [Source:HGNC Symbol;Acc:HGNC:24818]
ENSG00000128567	PODXL	-2,86274	podocalyxin like [Source:HGNC Symbol;Acc:HGNC:9171]
ENSG00000163812	ZDHHC3	2,859447	zinc finger DHHC-type containing 3 [Source:HGNC Symbol;Acc:HGNC:18470]
ENSG00000240476	LINC00973	2,858128	long intergenic non-protein coding RNA 973 [Source:HGNC Symbol;Acc:HGNC:48868]
ENSG00000167671	UBXN6	2,854637	UBX domain protein 6 [Source:HGNC Symbol;Acc:HGNC:14928]
ENSG00000204789	ZNF204P	2,853256	zinc finger protein 204, pseudogene [Source:HGNC Symbol;Acc:HGNC:12995]
ENSG00000138738	PRDM5	2,8532	PR/SET domain 5 [Source:HGNC Symbol;Acc:HGNC:9349]
ENSG00000242612	DECR2	-2,8477	2,4-dienoyl-CoA reductase 2 [Source:HGNC Symbol;Acc:HGNC:2754]
ENSG00000175984	DENND2C	2,844484	DENN domain containing 2C [Source:HGNC Symbol;Acc:HGNC:24748]
ENSG00000165886	UBTD1	2,842611	ubiquitin domain containing 1 [Source:HGNC Symbol;Acc:HGNC:25683]
ENSG00000163512	AZI2	2,837325	5-azacytidine induced 2 [Source:HGNC Symbol;Acc:HGNC:24002]
ENSG00000133069	TMCC2	2,836082	transmembrane and coiled-coil domain family 2 [Source:HGNC Symbol;Acc:HGNC:24239]
ENSG00000070610	GBA2	2,833408	glucosylceramidase beta 2 [Source:HGNC Symbol;Acc:HGNC:18986]
ENSG00000226846	LINC00348	-2,82892	long intergenic non-protein coding RNA 348 [Source:HGNC Symbol;Acc:HGNC:42658]
ENSG00000141084	RANBP10	2,815126	RAN binding protein 10 [Source:HGNC Symbol;Acc:HGNC:29285]
ENSG00000225377	NRSN2-AS1	-2,7962	NRSN2 antisense RNA 1 [Source:HGNC Symbol;Acc:HGNC:51222]
ENSG00000169914	OTUD3	2,79156	OTU deubiquitinase 3 [Source:HGNC Symbol;Acc:HGNC:29038]
ENSG00000111729	CLEC4A	2,787832	C-type lectin domain family 4 member A [Source:HGNC Symbol;Acc:HGNC:13257]
ENSG00000196433	ASMT	-2,78749	acetylserotonin O-methyltransferase [Source:HGNC Symbol;Acc:HGNC:750]
ENSG00000075413	MARK3	2,785411	microtubule affinity regulating kinase 3 [Source:HGNC Symbol;Acc:HGNC:6897]
ENSG00000134444	KIAA1468	2,782867	KIAA1468 [Source:HGNC Symbol;Acc:HGNC:29289]
ENSG00000198625	MDM4	2,776365	MDM4, p53 regulator [Source:HGNC Symbol;Acc:HGNC:6974]

ENSG00000142511	GPR32	-2,74158	G protein-coupled receptor 32 [Source:HGNC Symbol;Acc:HGNC:4487]
ENSG00000105550	FGF21	-2,73307	fibroblast growth factor 21 [Source:HGNC Symbol;Acc:HGNC:3678]
ENSG00000182606	TRAK1	2,73008	trafficking kinesin protein 1 [Source:HGNC Symbol;Acc:HGNC:29947]
ENSG00000235008		-2,72993	
ENSG00000180304	OAZ2	2,725851	ornithine decarboxylase antizyme 2 [Source:HGNC Symbol;Acc:HGNC:8096]
ENSG00000129355	CDKN2D	2,719689	cyclin dependent kinase inhibitor 2D [Source:HGNC Symbol;Acc:HGNC:1790]
ENSG00000101346	POFUT1	-2,71952	protein O-fucosyltransferase 1 [Source:HGNC Symbol;Acc:HGNC:14988]
ENSG00000100614	PPM1A	2,717327	protein phosphatase, Mg2+/Mn2+ dependent 1A [Source:HGNC Symbol;Acc:HGNC:9275]
ENSG00000154822	PLCL2	2,710696	phospholipase C like 2 [Source:HGNC Symbol;Acc:HGNC:9064]
ENSG00000238098	ABCA17P	-2,70513	ATP binding cassette subfamily A member 17, pseudogene [Source:HGNC Symbol;Acc:HGNC:32972]
ENSG00000175931	UBE2O	2,704673	ubiquitin conjugating enzyme E2 O [Source:HGNC Symbol;Acc:HGNC:29554]
ENSG00000104848	KCNA7	2,703474	potassium voltage-gated channel subfamily A member 7 [Source:HGNC Symbol;Acc:HGNC:6226]
ENSG00000203601	LINC00970	2,699312	long intergenic non-protein coding RNA 970 [Source:HGNC Symbol;Acc:HGNC:48730]
ENSG00000143303	RRNAD1	2,696598	ribosomal RNA adenine dimethylase domain containing 1 [Source:HGNC Symbol;Acc:HGNC:24273]
ENSG00000088826	SMOX	2,69163	spermine oxidase [Source:HGNC Symbol;Acc:HGNC:15862]
ENSG00000149716	ORAOV1	2,680456	oral cancer overexpressed 1 [Source:HGNC Symbol;Acc:HGNC:17589]
ENSG00000254082	NA	-2,67916	NA
ENSG00000117616	RSRP1	2,679137	arginine and serine rich protein 1 [Source:HGNC Symbol;Acc:HGNC:25234]
ENSG00000145416	MARCH1	2,678211	membrane associated ring-CH-type finger 1 [Source:HGNC Symbol;Acc:HGNC:26077]
ENSG00000022840	RNF10	2,674946	ring finger protein 10 [Source:HGNC Symbol;Acc:HGNC:10055]
ENSG00000135766	EGLN1	2,673763	egl-9 family hypoxia inducible factor 1 [Source:HGNC Symbol;Acc:HGNC:1232]
ENSG00000176714	CCDC121	-2,66861	coiled-coil domain containing 121 [Source:HGNC Symbol;Acc:HGNC:25833]
ENSG00000160131	VMA21	-2,66647	VMA21, vacuolar ATPase assembly factor [Source:HGNC Symbol;Acc:HGNC:22082]
ENSG00000139668	WDFY2	2,663186	WD repeat and FYVE domain containing 2 [Source:HGNC Symbol;Acc:HGNC:20482]
ENSG00000100364	KIAA0930	2,661839	KIAA0930 [Source:HGNC Symbol;Acc:HGNC:1314]
ENSG00000232103	NA	-2,65952	NA
ENSG00000171603	CLSTN1	-2,65158	calsyntenin 1 [Source:HGNC Symbol;Acc:HGNC:17447]
ENSG00000125971	DYNLRB1	-2,6468	dynein light chain roadblock-type 1 [Source:HGNC Symbol;Acc:HGNC:15468]
ENSG00000125965	GDF5	-2,64135	growth differentiation factor 5 [Source:HGNC Symbol;Acc:HGNC:4220]
ENSG00000105698	USF2	2,639631	upstream transcription factor 2, c-fos interacting [Source:HGNC Symbol;Acc:HGNC:12594]
ENSG00000227456	LINC00310	2,638308	long intergenic non-protein coding RNA 310 [Source:HGNC Symbol;Acc:HGNC:16414]

**Biological theme:**	**Ras-MAP kinase signalling cascade**
**Gene sets:**	PLCG1 events in ERBB2 signaling (Reactome)
	EGFR interacts with PLCG1 (Reactome)
	DAG and IP3 signaling (Reactome)
	NGF receptor signaling pathway (GO)
	NGF signalling via TRKA from the plasma membrane (Reactome)
	MAP2K1 subnetwork (ENSG)
	TGOLN2 subnetwork (ENSG)
	Downstream signal transduction (Reactome)
	Signalling by NGF (Reactome)
	CEBPB subnetwork (ENSG)
	CTNNB1 subnetwork (ENSG)
	Acute myeloid leukemia (KEGG)
	RAF1 subnetwork (ENSG)
	Non-small cell lung cancer (KEGG)

Ensembl gene ID	Gene	Weighted mean *z* -score	Description
ENSG00000105701	FKBP8	3,362565	FK506 binding protein 8 [Source:HGNC Symbol;Acc:HGNC:3724]
ENSG00000172354	GNB2	3,349456	G protein subunit beta 2 [Source:HGNC Symbol;Acc:HGNC:4398]
ENSG00000126767	ELK1	3,04926	ELK1, ETS transcription factor [Source:HGNC Symbol;Acc:HGNC:3321]
ENSG00000072518	MARK2	2,863907	microtubule affinity regulating kinase 2 [Source:HGNC Symbol;Acc:HGNC:3332]
ENSG00000174903	RAB1B	2,829124	RAB1B, member RAS oncogene family [Source:HGNC Symbol;Acc:HGNC:18370]
ENSG00000160113	NR2F6	2,783683	nuclear receptor subfamily 2 group F member 6 [Source:HGNC Symbol;Acc:HGNC:7977]
ENSG00000112576	CCND3	2,775147	cyclin D3 [Source:HGNC Symbol;Acc:HGNC:1585]
ENSG00000078369	GNB1	2,690021	G protein subunit beta 1 [Source:HGNC Symbol;Acc:HGNC:4396]
ENSG00000103495	MAZ	2,687244	MYC associated zinc finger protein [Source:HGNC Symbol;Acc:HGNC:6914]
ENSG00000148341	SH3GLB2	2,669614	SH3 domain containing GRB2 like, endophilin B2 [Source:HGNC Symbol;Acc:HGNC:10834]
ENSG00000088256	GNA11	2,66894	G protein subunit alpha 11 [Source:HGNC Symbol;Acc:HGNC:4379]
ENSG00000130311	DDA1	2,658982	DET1 and DDB1 associated 1 [Source:HGNC Symbol;Acc:HGNC:28360]
ENSG00000107929	LARP4B	2,658797	La ribonucleoprotein domain family member 4B [Source:HGNC Symbol;Acc:HGNC:28987]
ENSG00000173020	GRK2	2,64296	G protein-coupled receptor kinase 2 [Source:HGNC Symbol;Acc:HGNC:289]
ENSG00000105568	PPP2R1A	2,629801	protein phosphatase 2 scaffold subunit Aalpha [Source:HGNC Symbol;Acc:HGNC:9302]
ENSG00000014216	CAPN1	2,629618	calpain 1 [Source:HGNC Symbol;Acc:HGNC:1476]
ENSG00000104964	AES	2,585691	amino-terminal enhancer of split [Source:HGNC Symbol;Acc:HGNC:307]
ENSG00000187838	PLSCR3	2,579722	phospholipid scramblase 3 [Source:HGNC Symbol;Acc:HGNC:16495]
ENSG00000106367	AP1S1	2,575291	adaptor related protein complex 1 sigma 1 subunit [Source:HGNC Symbol;Acc:HGNC:559]
ENSG00000141522	ARHGDIA	2,570773	Rho GDP dissociation inhibitor alpha [Source:HGNC Symbol;Acc:HGNC:678]

ENSG00000130725	UBE2M	2,547863	ubiquitin conjugating enzyme E2 M [Source:HGNC Symbol;Acc:HGNC:12491]
ENSG00000119487	MAPKAP1	2,547659	mitogen-activated protein kinase associated protein 1 [Source:HGNC Symbol;Acc:HGNC:18752]
ENSG00000128989	ARPP19	2,52377	cAMP regulated phosphoprotein 19 [Source:HGNC Symbol;Acc:HGNC:16967]
ENSG00000169410	PTPN9	2,502804	protein tyrosine phosphatase, non-receptor type 9 [Source:HGNC Symbol;Acc:HGNC:9661]
ENSG00000079805	DNM2	2,501621	dynamin 2 [Source:HGNC Symbol;Acc:HGNC:2974]
ENSG00000048740	CELF2	2,500813	CUGBP Elav-like family member 2 [Source:HGNC Symbol;Acc:HGNC:2550]
ENSG00000156052	GNAQ	2,483294	G protein subunit alpha q [Source:HGNC Symbol;Acc:HGNC:4390]
ENSG00000172531	PPP1CA	2,483062	protein phosphatase 1 catalytic subunit alpha [Source:HGNC Symbol;Acc:HGNC:9281]
ENSG00000133243	BTBD2	2,481601	BTB domain containing 2 [Source:HGNC Symbol;Acc:HGNC:15504]
ENSG00000165807	PPP1R36	-2,47004	protein phosphatase 1 regulatory subunit 36 [Source:HGNC Symbol;Acc:HGNC:20097]
ENSG00000079335	CDC14A	2,458279	cell division cycle 14A [Source:HGNC Symbol;Acc:HGNC:1718]
ENSG00000102225	CDK16	2,453495	cyclin dependent kinase 16 [Source:HGNC Symbol;Acc:HGNC:8749]
ENSG00000169926	KLF13	2,450178	Kruppel like factor 13 [Source:HGNC Symbol;Acc:HGNC:13672]
ENSG00000114353	GNAI2	2,433884	G protein subunit alpha i2 [Source:HGNC Symbol;Acc:HGNC:4385]
ENSG00000065923	SLC9A7	2,433103	solute carrier family 9 member A7 [Source:HGNC Symbol;Acc:HGNC:17123]
ENSG00000155868	MED7	-2,4319	mediator complex subunit 7 [Source:HGNC Symbol;Acc:HGNC:2378]
ENSG00000175215	CTDSP2	2,424457	CTD small phosphatase 2 [Source:HGNC Symbol;Acc:HGNC:17077]
ENSG00000185236	RAB11B	2,423842	RAB11B, member RAS oncogene family [Source:HGNC Symbol;Acc:HGNC:9761]
ENSG00000063245	EPN1	2,418201	epsin 1 [Source:HGNC Symbol;Acc:HGNC:21604]
ENSG00000196588	MKL1	2,417129	megakaryoblastic leukemia (translocation) 1 [Source:HGNC Symbol;Acc:HGNC:14334]
ENSG00000213923	CSNK1E	2,395432	casein kinase 1 epsilon [Source:HGNC Symbol;Acc:HGNC:2453]
ENSG00000112078	KCTD20	2,391365	potassium channel tetramerization domain containing 20 [Source:HGNC Symbol;Acc:HGNC:21052]
ENSG00000123159	GIPC1	2,391234	GIPC PDZ domain containing family member 1 [Source:HGNC Symbol;Acc:HGNC:1226]
ENSG00000143498	TAF1A	-2,38313	TATA-box binding protein associated factor, RNA polymerase I subunit A [Source:HGNC Symbol;Acc:HGNC:11532]
ENSG00000156639	ZFAND3	2,373954	zinc finger AN1-type containing 3 [Source:HGNC Symbol;Acc:HGNC:18019]
ENSG00000104960	PTOV1	2,369452	prostate tumor overexpressed 1 [Source:HGNC Symbol;Acc:HGNC:9632]
ENSG00000141551	CSNK1D	2,351336	casein kinase 1 delta [Source:HGNC Symbol;Acc:HGNC:2452]
ENSG00000135932	CAB39	2,351296	calcium binding protein 39 [Source:HGNC Symbol;Acc:HGNC:20292]
ENSG00000119929	CUTC	-2,32744	cutC copper transporter [Source:HGNC Symbol;Acc:HGNC:24271]
ENSG00000131408	NR1H2	2,32513	nuclear receptor subfamily 1 group H member 2 [Source:HGNC Symbol;Acc:HGNC:7965]
ENSG00000130511	SSBP4	2,317372	single stranded DNA binding protein 4 [Source:HGNC Symbol;Acc:HGNC:15676]
ENSG00000062598	ELMO2	2,314481	engulfment and cell motility 2 [Source:HGNC Symbol;Acc:HGNC:17233]
ENSG00000163376	KBTBD8	-2,31382	kelch repeat and BTB domain containing 8 [Source:HGNC Symbol;Acc:HGNC:30691]
ENSG00000177565	TBL1XR1	2,304209	transducin beta like 1 X-linked receptor 1 [Source:HGNC Symbol;Acc:HGNC:29529]
ENSG00000172995	ARPP21	2,300644	cAMP regulated phosphoprotein 21 [Source:HGNC Symbol;Acc:HGNC:16968]
ENSG00000055917	PUM2	2,299191	pumilio RNA binding family member 2 [Source:HGNC Symbol;Acc:HGNC:14958]
ENSG00000138814	PPP3CA	2,288783	protein phosphatase 3 catalytic subunit alpha [Source:HGNC Symbol;Acc:HGNC:9314]

ENSG00000149091	DGKZ	2,28256	diacylglycerol kinase zeta [Source:HGNC Symbol;Acc:HGNC:2857]
ENSG00000090372	STRN4	2,275145	striatin 4 [Source:HGNC Symbol;Acc:HGNC:15721]
ENSG00000198909	MAP3K3	2,263167	mitogen-activated protein kinase kinase kinase 3 [Source:HGNC Symbol;Acc:HGNC:6855]
ENSG00000100241	SBF1	2,26085	SET binding factor 1 [Source:HGNC Symbol;Acc:HGNC:10542]
ENSG00000158985	CDC42SE2	2,25784	CDC42 small effector 2 [Source:HGNC Symbol;Acc:HGNC:18547]
ENSG00000065491	TBC1D22B	2,252824	TBC1 domain family member 22B [Source:HGNC Symbol;Acc:HGNC:21602]
ENSG00000087274	ADD1	2,250897	adducin 1 [Source:HGNC Symbol;Acc:HGNC:243]
ENSG00000129911	KLF16	2,245994	Kruppel like factor 16 [Source:HGNC Symbol;Acc:HGNC:16857]
ENSG00000120063	GNA13	2,24276	G protein subunit alpha 13 [Source:HGNC Symbol;Acc:HGNC:4381]
ENSG00000124151	NCOA3	2,240209	nuclear receptor coactivator 3 [Source:HGNC Symbol;Acc:HGNC:7670]
ENSG00000138594	TMOD3	2,220182	tropomodulin 3 [Source:HGNC Symbol;Acc:HGNC:11873]
ENSG00000157014	TATDN2	2,218306	TatD DNase domain containing 2 [Source:HGNC Symbol;Acc:HGNC:28988]
ENSG00000172081	MOB3A	2,218104	MOB kinase activator 3A [Source:HGNC Symbol;Acc:HGNC:29802]
ENSG00000163466	ARPC2	2,216147	actin related protein 2/3 complex subunit 2 [Source:HGNC Symbol;Acc:HGNC:705]
ENSG00000167323	STIM1	2,213045	stromal interaction molecule 1 [Source:HGNC Symbol;Acc:HGNC:11386]
ENSG00000125249	RAP2A	2,208114	RAP2A, member of RAS oncogene family [Source:HGNC Symbol;Acc:HGNC:9861]
ENSG00000166068	SPRED1	2,207657	sprouty related EVH1 domain containing 1 [Source:HGNC Symbol;Acc:HGNC:20249]
ENSG00000149212	SESN3	2,207497	sestrin 3 [Source:HGNC Symbol;Acc:HGNC:23060]
ENSG00000105647	PIK3R2	2,205978	phosphoinositide-3-kinase regulatory subunit 2 [Source:HGNC Symbol;Acc:HGNC:8980]
ENSG00000130045	NXNL2	-2,20034	nucleoredoxin-like 2 [Source:HGNC Symbol;Acc:HGNC:30482]
ENSG00000105325	FZR1	2,190648	fizzy and cell division cycle 20 related 1 [Source:HGNC Symbol;Acc:HGNC:24824]
ENSG00000123143	PKN1	2,190442	protein kinase N1 [Source:HGNC Symbol;Acc:HGNC:9405]
ENSG00000172575	RASGRP1	2,185034	RAS guanyl releasing protein 1 [Source:HGNC Symbol;Acc:HGNC:9878]
ENSG00000119383	PTPA	2,181049	protein phosphatase 2 phosphatase activator [Source:HGNC Symbol;Acc:HGNC:9308]
ENSG00000107263	RAPGEF1	2,180999	Rap guanine nucleotide exchange factor 1 [Source:HGNC Symbol;Acc:HGNC:4568]
ENSG00000160445	ZER1	2,174187	zyg-11 related cell cycle regulator [Source:HGNC Symbol;Acc:HGNC:30960]
ENSG00000149483	TMEM138	-2,17138	transmembrane protein 138 [Source:HGNC Symbol;Acc:HGNC:26944]
ENSG00000112664	NA	2,171116	NA
ENSG00000175416	CLTB	2,164399	clathrin light chain B [Source:HGNC Symbol;Acc:HGNC:2091]
ENSG00000196821	C6orf106	2,164271	chromosome 6 open reading frame 106 [Source:HGNC Symbol;Acc:HGNC:21215]
ENSG00000126214	KLC1	2,141003	kinesin light chain 1 [Source:HGNC Symbol;Acc:HGNC:6387]
ENSG00000196961	AP2A1	2,1407	adaptor related protein complex 2 alpha 1 subunit [Source:HGNC Symbol;Acc:HGNC:561]
ENSG00000155903	RASA2	2,140517	RAS p21 protein activator 2 [Source:HGNC Symbol;Acc:HGNC:9872]
ENSG00000134996	OSTF1	2,138074	osteoclast stimulating factor 1 [Source:HGNC Symbol;Acc:HGNC:8510]
ENSG00000167264	DUS2	-2,13716	dihydrouridine synthase 2 [Source:HGNC Symbol;Acc:HGNC:26014]
ENSG00000120899	PTK2B	2,132218	protein tyrosine kinase 2 beta [Source:HGNC Symbol;Acc:HGNC:9612]
ENSG00000154001	PPP2R5E	2,131273	protein phosphatase 2 regulatory subunit B'epsilon [Source:HGNC Symbol;Acc:HGNC:9313]

ENSG00000071051	NCK2	2,126936	NCK adaptor protein 2 [Source:HGNC Symbol;Acc:HGNC:7665]
ENSG00000101940	WDR13	2,120402	WD repeat domain 13 [Source:HGNC Symbol;Acc:HGNC:14352]
ENSG00000182979	MTA1	2,117818	metastasis associated 1 [Source:HGNC Symbol;Acc:HGNC:7410]
ENSG00000127220	ABHD8	2,117654	abhydrolase domain containing 8 [Source:HGNC Symbol;Acc:HGNC:23759]
ENSG00000130544	ZNF557	-2,11487	zinc finger protein 557 [Source:HGNC Symbol;Acc:HGNC:28632]
ENSG00000134343	ANO3	2,109273	anoctamin 3 [Source:HGNC Symbol;Acc:HGNC:14004]

**Biological theme:**	**Metabolism and adipositas**
**Gene sets:**	Reduced female fertility (MP)
	Prolonged estrous cycle (MP)
	Increased percent body fat (MP)
	Abnormal osteoclast differentiation (MP)

Ensembl gene ID	Gene	Weighted mean *z* -score	Description
ENSG00000166819	PLIN1	4,700457	perilipin 1 [Source:HGNC Symbol;Acc:HGNC:9076]
ENSG00000123612	ACVR1C	3,520797	activin A receptor type 1C [Source:HGNC Symbol;Acc:HGNC:18123]
ENSG00000196616	ADH1B	3,472607	alcohol dehydrogenase 1B (class I), beta polypeptide [Source:HGNC Symbol;Acc:HGNC:250]
ENSG00000134531	EMP1	3,321761	epithelial membrane protein 1 [Source:HGNC Symbol;Acc:HGNC:3333]
ENSG00000187288	CIDEC	3,313229	cell death inducing DFFA like effector c [Source:HGNC Symbol;Acc:HGNC:24229]
ENSG00000137331	IER3	3,177386	immediate early response 3 [Source:HGNC Symbol;Acc:HGNC:5392]
ENSG00000157150	TIMP4	3,163388	TIMP metallopeptidase inhibitor 4 [Source:HGNC Symbol;Acc:HGNC:11823]
ENSG00000128342	LIF	3,132582	LIF, interleukin 6 family cytokine [Source:HGNC Symbol;Acc:HGNC:6596]
ENSG00000170323	FABP4	3,127135	fatty acid binding protein 4 [Source:HGNC Symbol;Acc:HGNC:3559]
ENSG00000138061	CYP1B1	3,117281	cytochrome P450 family 1 subfamily B member 1 [Source:HGNC Symbol;Acc:HGNC:2597]
ENSG00000181092	ADIPOQ	3,048448	adiponectin, C1Q and collagen domain containing [Source:HGNC Symbol;Acc:HGNC:13633]
ENSG00000175445	LPL	3,034297	lipoprotein lipase [Source:HGNC Symbol;Acc:HGNC:6677]
ENSG00000108702	CCL1	3,033223	C-C motif chemokine ligand 1 [Source:HGNC Symbol;Acc:HGNC:10609]
ENSG00000144802	NFKBIZ	3,023706	NFKB inhibitor zeta [Source:HGNC Symbol;Acc:HGNC:29805]
ENSG00000185022	MAFF	3,018438	MAF bZIP transcription factor F [Source:HGNC Symbol;Acc:HGNC:6780]
ENSG00000167676	PLIN4	2,972947	perilipin 4 [Source:HGNC Symbol;Acc:HGNC:29393]
ENSG00000079435	LIPE	2,953482	lipase E, hormone sensitive type [Source:HGNC Symbol;Acc:HGNC:6621]
ENSG00000233093	LINC00892	2,937532	long intergenic non-protein coding RNA 892 [Source:HGNC Symbol;Acc:HGNC:48578]
ENSG00000173198	CYSLTR1	2,902014	cysteinyl leukotriene receptor 1 [Source:HGNC Symbol;Acc:HGNC:17451]
ENSG00000184371	CSF1	2,900313	colony stimulating factor 1 [Source:HGNC Symbol;Acc:HGNC:2432]
ENSG00000133317	LGALS12	2,884047	galectin 12 [Source:HGNC Symbol;Acc:HGNC:15788]
ENSG00000112964	GHR	2,875365	growth hormone receptor [Source:HGNC Symbol;Acc:HGNC:4263]
ENSG00000124253	PCK1	2,863389	phosphoenolpyruvate carboxykinase 1 [Source:HGNC Symbol;Acc:HGNC:8724]
ENSG00000221869	CEBPD	2,851151	CCAAT/enhancer binding protein delta [Source:HGNC Symbol;Acc:HGNC:1835]
ENSG00000159388	BTG2	2,844363	BTG anti-proliferation factor 2 [Source:HGNC Symbol;Acc:HGNC:1131]
ENSG00000100906	NFKBIA	2,812245	NFKB inhibitor alpha [Source:HGNC Symbol;Acc:HGNC:7797]
ENSG00000113389	NPR3	2,805784	natriuretic peptide receptor 3 [Source:HGNC Symbol;Acc:HGNC:7945]
ENSG00000073756	PTGS2	2,800178	prostaglandin-endoperoxide synthase 2 [Source:HGNC Symbol;Acc:HGNC:9605]
ENSG00000169194	IL13	2,763667	interleukin 13 [Source:HGNC Symbol;Acc:HGNC:5973]
ENSG00000167588	GPD1	2,761998	glycerol-3-phosphate dehydrogenase 1 [Source:HGNC Symbol;Acc:HGNC:4455]

ENSG00000138207	RBP4	2,751194	retinol binding protein 4 [Source:HGNC Symbol;Acc:HGNC:9922]
ENSG00000104043	ATP8B4	2,749891	ATPase phospholipid transporting 8B4 (putative) [Source:HGNC Symbol;Acc:HGNC:13536]
ENSG00000169429	CXCL8	2,721233	C-X-C motif chemokine ligand 8 [Source:HGNC Symbol;Acc:HGNC:6025]
ENSG00000176194	CIDEA	2,718866	cell death-inducing DFFA-like effector a [Source:HGNC Symbol;Acc:HGNC:1976]
ENSG00000178789	CD300LB	2,714743	CD300 molecule like family member b [Source:HGNC Symbol;Acc:HGNC:30811]
ENSG00000108960	MMD	2,702595	monocyte to macrophage differentiation associated [Source:HGNC Symbol;Acc:HGNC:7153]
ENSG00000116711	PLA2G4A	2,698043	phospholipase A2 group IVA [Source:HGNC Symbol;Acc:HGNC:9035]
ENSG00000119508	NR4A3	2,686126	nuclear receptor subfamily 4 group A member 3 [Source:HGNC Symbol;Acc:HGNC:7982]
ENSG00000101938	CHRDL1	2,685505	chordin like 1 [Source:HGNC Symbol;Acc:HGNC:29861]
ENSG00000148339	SLC25A25	2,680769	solute carrier family 25 member 25 [Source:HGNC Symbol;Acc:HGNC:20663]
ENSG00000108551	RASD1	2,674321	ras related dexamethasone induced 1 [Source:HGNC Symbol;Acc:HGNC:15828]
ENSG00000135218	CD36	2,64047	CD36 molecule [Source:HGNC Symbol;Acc:HGNC:1663]
ENSG00000164949	GEM	2,637479	GTP binding protein overexpressed in skeletal muscle [Source:HGNC Symbol;Acc:HGNC:4234]
ENSG00000125740	FOSB	2,636002	FosB proto-oncogene, AP-1 transcription factor subunit [Source:HGNC Symbol;Acc:HGNC:3797]
ENSG00000128016	ZFP36	2,623423	ZFP36 ring finger protein [Source:HGNC Symbol;Acc:HGNC:12862]
ENSG00000050628	PTGER3	2,600438	prostaglandin E receptor 3 [Source:HGNC Symbol;Acc:HGNC:9595]
ENSG00000138135	CH25H	2,598787	cholesterol 25-hydroxylase [Source:HGNC Symbol;Acc:HGNC:1907]
ENSG00000171223	JUNB	2,584347	JunB proto-oncogene, AP-1 transcription factor subunit [Source:HGNC Symbol;Acc:HGNC:6205]
ENSG00000184811	TUSC5	2,578702	tumor suppressor candidate 5 [Source:HGNC Symbol;Acc:HGNC:29592]
ENSG00000137462	TLR2	2,569844	toll like receptor 2 [Source:HGNC Symbol;Acc:HGNC:11848]
ENSG00000188783	PRELP	2,559681	proline and arginine rich end leucine rich repeat protein [Source:HGNC Symbol;Acc:HGNC:9357]
ENSG00000105825	TFPI2	2,55553	tissue factor pathway inhibitor 2 [Source:HGNC Symbol;Acc:HGNC:11761]
ENSG00000164400	CSF2	2,546836	colony stimulating factor 2 [Source:HGNC Symbol;Acc:HGNC:2434]
ENSG00000197766	CFD	2,544783	complement factor D [Source:HGNC Symbol;Acc:HGNC:2771]
ENSG00000081041	CXCL2	2,534	C-X-C motif chemokine ligand 2 [Source:HGNC Symbol;Acc:HGNC:4603]
ENSG00000163734	CXCL3	2,530983	C-X-C motif chemokine ligand 3 [Source:HGNC Symbol;Acc:HGNC:4604]
ENSG00000106823	ECM2	2,527061	extracellular matrix protein 2 [Source:HGNC Symbol;Acc:HGNC:3154]
ENSG00000130876	SLC7A10	2,523413	solute carrier family 7 member 10 [Source:HGNC Symbol;Acc:HGNC:11058]
ENSG00000069702	TGFBR3	2,516274	transforming growth factor beta receptor 3 [Source:HGNC Symbol;Acc:HGNC:11774]
ENSG00000143507	DUSP10	2,488437	dual specificity phosphatase 10 [Source:HGNC Symbol;Acc:HGNC:3065]
ENSG00000120738	EGR1	2,478584	early growth response 1 [Source:HGNC Symbol;Acc:HGNC:3238]
ENSG00000129596	CDO1	2,475957	cysteine dioxygenase type 1 [Source:HGNC Symbol;Acc:HGNC:1795]
ENSG00000163823	CCR1	2,470725	C-C motif chemokine receptor 1 [Source:HGNC Symbol;Acc:HGNC:1602]
ENSG00000118515	SGK1	2,460405	serum/glucocorticoid regulated kinase 1 [Source:HGNC Symbol;Acc:HGNC:10810]
ENSG00000106178	CCL24	2,458166	C-C motif chemokine ligand 24 [Source:HGNC Symbol;Acc:HGNC:10623]
ENSG00000123243	ITIH5	2,449515	inter-alpha-trypsin inhibitor heavy chain family member 5 [Source:HGNC Symbol;Acc:HGNC:21449]
ENSG00000108688	CCL7	2,449285	C-C motif chemokine ligand 7 [Source:HGNC Symbol;Acc:HGNC:10634]

ENSG00000163874	ZC3H12A	2,448402	zinc finger CCCH-type containing 12A [Source:HGNC Symbol;Acc:HGNC:26259]
ENSG00000136826	KLF4	2,441602	Kruppel like factor 4 [Source:HGNC Symbol;Acc:HGNC:6348]
ENSG00000056998	GYG2	2,441546	glycogenin 2 [Source:HGNC Symbol;Acc:HGNC:4700]
ENSG00000134070	IRAK2	2,42997	interleukin 1 receptor associated kinase 2 [Source:HGNC Symbol;Acc:HGNC:6113]
ENSG00000196917	HCAR1	2,425992	hydroxycarboxylic acid receptor 1 [Source:HGNC Symbol;Acc:HGNC:4532]
ENSG00000158691	ZSCAN12	-2,42346	zinc finger and SCAN domain containing 12 [Source:HGNC Symbol;Acc:HGNC:13172]
ENSG00000158571	PFKFB1	2,394879	6-phosphofructo-2-kinase/fructose-2,6-biphosphatase 1 [Source:HGNC Symbol;Acc:HGNC:8872]
ENSG00000102760	RGCC	2,393504	regulator of cell cycle [Source:HGNC Symbol;Acc:HGNC:20369]
ENSG00000173114	LRRN3	2,39204	leucine rich repeat neuronal 3 [Source:HGNC Symbol;Acc:HGNC:17200]
ENSG00000162772	ATF3	2,390251	activating transcription factor 3 [Source:HGNC Symbol;Acc:HGNC:785]
ENSG00000170525	PFKFB3	2,39	6-phosphofructo-2-kinase/fructose-2,6-biphosphatase 3 [Source:HGNC Symbol;Acc:HGNC:8874]
ENSG00000184601	C14orf180	2,389943	chromosome 14 open reading frame 180 [Source:HGNC Symbol;Acc:HGNC:33795]
ENSG00000177666	PNPLA2	2,38564	patatin like phospholipase domain containing 2 [Source:HGNC Symbol;Acc:HGNC:30802]
ENSG00000127955	GNAI1	2,377172	G protein subunit alpha i1 [Source:HGNC Symbol;Acc:HGNC:4384]
ENSG00000153234	NR4A2	2,35997	nuclear receptor subfamily 4 group A member 2 [Source:HGNC Symbol;Acc:HGNC:7981]
ENSG00000107104	KANK1	2,359699	KN motif and ankyrin repeat domains 1 [Source:HGNC Symbol;Acc:HGNC:19309]
ENSG00000106809	OGN	2,359074	osteoglycin [Source:HGNC Symbol;Acc:HGNC:8126]
ENSG00000113520	IL4	2,358315	interleukin 4 [Source:HGNC Symbol;Acc:HGNC:6014]
ENSG00000152315	KCNK13	2,35296	potassium two pore domain channel subfamily K member 13 [Source:HGNC Symbol;Acc:HGNC:6275]
ENSG00000164129	NPY5R	2,339855	neuropeptide Y receptor Y5 [Source:HGNC Symbol;Acc:HGNC:7958]
ENSG00000028137	TNFRSF1B	2,338504	TNF receptor superfamily member 1B [Source:HGNC Symbol;Acc:HGNC:11917]
ENSG00000107968	MAP3K8	2,336486	mitogen-activated protein kinase kinase kinase 8 [Source:HGNC Symbol;Acc:HGNC:6860]
ENSG00000151090	THRB	2,331705	thyroid hormone receptor beta [Source:HGNC Symbol;Acc:HGNC:11799]
ENSG00000134107	BHLHE40	2,329507	basic helix-loop-helix family member e40 [Source:HGNC Symbol;Acc:HGNC:1046]
ENSG00000115009	CCL20	2,320541	C-C motif chemokine ligand 20 [Source:HGNC Symbol;Acc:HGNC:10619]
ENSG00000152270	PDE3B	2,31417	phosphodiesterase 3B [Source:HGNC Symbol;Acc:HGNC:8779]
ENSG00000173846	PLK3	2,308781	polo like kinase 3 [Source:HGNC Symbol;Acc:HGNC:2154]
ENSG00000135447	PPP1R1A	2,305536	protein phosphatase 1 regulatory inhibitor subunit 1A [Source:HGNC Symbol;Acc:HGNC:9286]
ENSG00000184557	SOCS3	2,30321	suppressor of cytokine signaling 3 [Source:HGNC Symbol;Acc:HGNC:19391]
ENSG00000171659	GPR34	2,297329	G protein-coupled receptor 34 [Source:HGNC Symbol;Acc:HGNC:4490]
ENSG00000170458	CD14	2,296518	CD14 molecule [Source:HGNC Symbol;Acc:HGNC:1628]
ENSG00000125735	TNFSF14	2,2948	TNF superfamily member 14 [Source:HGNC Symbol;Acc:HGNC:11930]
ENSG00000119121	TRPM6	2,271932	transient receptor potential cation channel subfamily M member 6 [Source:HGNC Symbol;Acc:HGNC:17995]

**Biological theme:**	**Signaling by EGFR**
**Gene sets:**	PLCG1 events in ERBB2 signaling (Reactome)
	EGFR interacts with PLCG1 (Reactome)
	DAG and IP3 signaling (Reactome)

**Ensembl gene ID**	**Gene**	**Weighted mean *z* -score**	**Description**
ENSG00000249601	LINC01187	4,38077	long intergenic non-protein coding RNA 1187 [Source:HGNC Symbol;Acc:HGNC:49575]
ENSG00000184908	CLCNKB	4,217502	chloride voltage-gated channel Kb [Source:HGNC Symbol;Acc:HGNC:2027]
ENSG00000161381	PLXDC1	4,123238	plexin domain containing 1 [Source:HGNC Symbol;Acc:HGNC:20945]
ENSG00000182732	RGS6	3,990554	regulator of G protein signaling 6 [Source:HGNC Symbol;Acc:HGNC:10002]
ENSG00000114861	FOXP1	3,943525	forkhead box P1 [Source:HGNC Symbol;Acc:HGNC:3823]
ENSG00000086159	AQP6	3,9232	aquaporin 6 [Source:HGNC Symbol;Acc:HGNC:639]
ENSG00000152642	GPD1L	3,915456	glycerol-3-phosphate dehydrogenase 1-like [Source:HGNC Symbol;Acc:HGNC:28956]
ENSG00000124772	CPNE5	3,894899	copine 5 [Source:HGNC Symbol;Acc:HGNC:2318]
ENSG00000154146	NRGN	3,884946	neurogranin [Source:HGNC Symbol;Acc:HGNC:8000]
ENSG00000168269	FOXI1	3,882321	forkhead box I1 [Source:HGNC Symbol;Acc:HGNC:3815]
ENSG00000143473	KCNH1	3,876158	potassium voltage-gated channel subfamily H member 1 [Source:HGNC Symbol;Acc:HGNC:6250]
ENSG00000123612	ACVR1C	3,843504	activin A receptor type 1C [Source:HGNC Symbol;Acc:HGNC:18123]
ENSG00000183146	PRORY	3,694305	proline rich, Y-linked [Source:HGNC Symbol;Acc:HGNC:38732]
ENSG00000078687	TNRC6C	3,687856	trinucleotide repeat containing 6C [Source:HGNC Symbol;Acc:HGNC:29318]
ENSG00000184845	DRD1	3,654582	dopamine receptor D1 [Source:HGNC Symbol;Acc:HGNC:3020]
ENSG00000065989	PDE4A	3,65352	phosphodiesterase 4A [Source:HGNC Symbol;Acc:HGNC:8780]
ENSG00000151418	ATP6V1G3	3,640113	ATPase H+ transporting V1 subunit G3 [Source:HGNC Symbol;Acc:HGNC:18265]
ENSG00000183454	GRIN2A	3,616126	glutamate ionotropic receptor NMDA type subunit 2A [Source:HGNC Symbol;Acc:HGNC:4585]
ENSG00000144596	GRIP2	3,575832	glutamate receptor interacting protein 2 [Source:HGNC Symbol;Acc:HGNC:23841]
ENSG00000231249	ITPR1-AS1	3,547294	ITPR1 antisense RNA 1 (head to head) [Source:HGNC Symbol;Acc:HGNC:44470]
ENSG00000083720	OXCT1	3,544402	3-oxoacid CoA-transferase 1 [Source:HGNC Symbol;Acc:HGNC:8527]
ENSG00000167693	NXN	-3,53507	nucleoredoxin [Source:HGNC Symbol;Acc:HGNC:18008]
ENSG00000137207	YIPF3	-3,50762	Yip1 domain family member 3 [Source:HGNC Symbol;Acc:HGNC:21023]
ENSG00000153012	LGI2	3,48333	leucine rich repeat LGI family member 2 [Source:HGNC Symbol;Acc:HGNC:18710]
ENSG00000243627		3,476747	
ENSG00000181656	GPR88	3,469888	G protein-coupled receptor 88 [Source:HGNC Symbol;Acc:HGNC:4539]
ENSG00000123119	NECAB1	3,4643	N-terminal EF-hand calcium binding protein 1 [Source:HGNC Symbol;Acc:HGNC:20983]
ENSG00000131067	GGT7	3,460942	gamma-glutamyltransferase 7 [Source:HGNC Symbol;Acc:HGNC:4259]
ENSG00000137486	ARRB1	3,451354	arrestin beta 1 [Source:HGNC Symbol;Acc:HGNC:711]
ENSG00000178567	EPM2AIP1	3,423901	EPM2A interacting protein 1 [Source:HGNC Symbol;Acc:HGNC:19735]
ENSG00000175471	MCTP1	3,380028	multiple C2 and transmembrane domain containing 1 [Source:HGNC Symbol;Acc:HGNC:26183]

ENSG00000109158	GABRA4	3,370186	gamma-aminobutyric acid type A receptor alpha4 subunit [Source:HGNC Symbol;Acc:HGNC:4078]
ENSG00000164076	CAMKV	3,352136	CaM kinase like vesicle associated [Source:HGNC Symbol;Acc:HGNC:28788]
ENSG00000248550	OTX2-AS1	-3,34859	OTX2 antisense RNA 1 (head to head) [Source:HGNC Symbol;Acc:HGNC:43906]
ENSG00000117868	ESYT2	3,339913	extended synaptotagmin 2 [Source:HGNC Symbol;Acc:HGNC:22211]
ENSG00000150672	DLG2	3,334791	discs large MAGUK scaffold protein 2 [Source:HGNC Symbol;Acc:HGNC:2901]
ENSG00000187800	PEAR1	3,331669	platelet endothelial aggregation receptor 1 [Source:HGNC Symbol;Acc:HGNC:33631]
ENSG00000186479	RGS7BP	3,329693	regulator of G protein signaling 7 binding protein [Source:HGNC Symbol;Acc:HGNC:23271]
ENSG00000185133	INPP5J	3,327221	inositol polyphosphate-5-phosphatase J [Source:HGNC Symbol;Acc:HGNC:8956]
ENSG00000154016	GRAP	3,323793	GRB2-related adaptor protein [Source:HGNC Symbol;Acc:HGNC:4562]
ENSG00000176884	GRIN1	3,310171	glutamate ionotropic receptor NMDA type subunit 1 [Source:HGNC Symbol;Acc:HGNC:4584]
ENSG00000151623	NR3C2	3,29812	nuclear receptor subfamily 3 group C member 2 [Source:HGNC Symbol;Acc:HGNC:7979]
ENSG00000172575	RASGRP1	3,297642	RAS guanyl releasing protein 1 [Source:HGNC Symbol;Acc:HGNC:9878]
ENSG00000172572	PDE3A	3,29653	phosphodiesterase 3A [Source:HGNC Symbol;Acc:HGNC:8778]
ENSG00000173258	ZNF483	3,28762	zinc finger protein 483 [Source:HGNC Symbol;Acc:HGNC:23384]
ENSG00000187527	ATP13A5	3,281594	ATPase 13A5 [Source:HGNC Symbol;Acc:HGNC:31789]
ENSG00000203685	STUM	3,265948	stum, mechanosensory transduction mediator homolog [Source:HGNC Symbol;Acc:HGNC:30491]
ENSG00000152270	PDE3B	3,253739	phosphodiesterase 3B [Source:HGNC Symbol;Acc:HGNC:8779]
ENSG00000249862	NA	-3,22401	NA
ENSG00000137500	CCDC90B	-3,2125	coiled-coil domain containing 90B [Source:HGNC Symbol;Acc:HGNC:28108]
ENSG00000175868	CALCB	-3,20405	calcitonin related polypeptide beta [Source:HGNC Symbol;Acc:HGNC:1438]
ENSG00000072952	MRVI1	3,190977	murine retrovirus integration site 1 homolog [Source:HGNC Symbol;Acc:HGNC:7237]
ENSG00000110427	KIAA1549L	3,18682	KIAA1549 like [Source:HGNC Symbol;Acc:HGNC:24836]
ENSG00000177098	SCN4B	3,179776	sodium voltage-gated channel beta subunit 4 [Source:HGNC Symbol;Acc:HGNC:10592]
ENSG00000114279	FGF12	3,166897	fibroblast growth factor 12 [Source:HGNC Symbol;Acc:HGNC:3668]
ENSG00000258343		3,158362	
ENSG00000162852	CNST	3,148927	consortin, connexin sorting protein [Source:HGNC Symbol;Acc:HGNC:26486]
ENSG00000150995	ITPR1	3,147663	inositol 1,4,5-trisphosphate receptor type 1 [Source:HGNC Symbol;Acc:HGNC:6180]
ENSG00000164100	NDST3	3,13959	N-deacetylase and N-sulfotransferase 3 [Source:HGNC Symbol;Acc:HGNC:7682]
ENSG00000249231	CASC16	3,133528	cancer susceptibility 16 (non-protein coding) [Source:HGNC Symbol;Acc:HGNC:48608]
ENSG00000134318	ROCK2	3,128455	Rho associated coiled-coil containing protein kinase 2 [Source:HGNC Symbol;Acc:HGNC:10252]
ENSG00000006638	TBXA2R	3,12627	thromboxane A2 receptor [Source:HGNC Symbol;Acc:HGNC:11608]
ENSG00000115488	NEU2	3,121715	neuraminidase 2 [Source:HGNC Symbol;Acc:HGNC:7759]
ENSG00000163629	PTPN13	-3,11982	protein tyrosine phosphatase, non-receptor type 13 [Source:HGNC Symbol;Acc:HGNC:9646]
ENSG00000138688	KIAA1109	3,119247	KIAA1109 [Source:HGNC Symbol;Acc:HGNC:26953]
ENSG00000090975	PITPNM2	3,115216	phosphatidylinositol transfer protein membrane associated 2 [Source:HGNC Symbol;Acc:HGNC:21044]
ENSG00000185924	RTN4RL1	3,101151	reticulon 4 receptor like 1 [Source:HGNC Symbol;Acc:HGNC:21329]
ENSG00000186642	PDE2A	3,100456	phosphodiesterase 2A [Source:HGNC Symbol;Acc:HGNC:8777]

ENSG00000171611	PTCRA	3,099628	pre T-cell antigen receptor alpha [Source:HGNC Symbol;Acc:HGNC:21290]
ENSG00000172379	ARNT2	3,098058	aryl hydrocarbon receptor nuclear translocator 2 [Source:HGNC Symbol;Acc:HGNC:16876]
ENSG00000145920	CPLX2	3,095933	complexin 2 [Source:HGNC Symbol;Acc:HGNC:2310]
ENSG00000149639	SOGA1	3,094996	suppressor of glucose, autophagy associated 1 [Source:HGNC Symbol;Acc:HGNC:16111]
ENSG00000179222	MAGED1	-3,09444	MAGE family member D1 [Source:HGNC Symbol;Acc:HGNC:6813]
ENSG00000231426		-3,0931	
ENSG00000069966	GNB5	3,079981	G protein subunit beta 5 [Source:HGNC Symbol;Acc:HGNC:4401]
ENSG00000102468	HTR2A	3,071402	5-hydroxytryptamine receptor 2A [Source:HGNC Symbol;Acc:HGNC:5293]
ENSG00000025156	HSF2	-3,0557	heat shock transcription factor 2 [Source:HGNC Symbol;Acc:HGNC:5225]
ENSG00000147408	CSGALNACT1	3,054878	chondroitin sulfate N-acetylgalactosaminyltransferase 1 [Source:HGNC Symbol;Acc:HGNC:24290]
ENSG00000053918	KCNQ1	3,053721	potassium voltage-gated channel subfamily Q member 1 [Source:HGNC Symbol;Acc:HGNC:6294]
ENSG00000197106	SLC6A17	3,049477	solute carrier family 6 member 17 [Source:HGNC Symbol;Acc:HGNC:31399]
ENSG00000168135	KCNJ4	3,049114	potassium voltage-gated channel subfamily J member 4 [Source:HGNC Symbol;Acc:HGNC:6265]
ENSG00000061337	LZTS1	3,048869	leucine zipper tumor suppressor 1 [Source:HGNC Symbol;Acc:HGNC:13861]
ENSG00000259207	ITGB3	3,040978	integrin subunit beta 3 [Source:HGNC Symbol;Acc:HGNC:6156]
ENSG00000102309	PIN4	-3,03536	peptidylprolyl cis/trans isomerase, NIMA-interacting 4 [Source:HGNC Symbol;Acc:HGNC:8992]
ENSG00000163491	NEK10	3,032945	NIMA related kinase 10 [Source:HGNC Symbol;Acc:HGNC:18592]
ENSG00000101190	TCFL5	3,024156	transcription factor like 5 [Source:HGNC Symbol;Acc:HGNC:11646]
ENSG00000157470	FAM81A	3,023168	family with sequence similarity 81 member A [Source:HGNC Symbol;Acc:HGNC:28379]
ENSG00000121057	AKAP1	3,021357	A-kinase anchoring protein 1 [Source:HGNC Symbol;Acc:HGNC:367]
ENSG00000124215	CDH26	-3,01493	cadherin 26 [Source:HGNC Symbol;Acc:HGNC:15902]
ENSG00000168818	STX18	-3,01412	syntaxin 18 [Source:HGNC Symbol;Acc:HGNC:15942]
ENSG00000145911	N4BP3	3,007796	NEDD4 binding protein 3 [Source:HGNC Symbol;Acc:HGNC:29852]
ENSG00000151693	ASAP2	2,999487	ArfGAP with SH3 domain, ankyrin repeat and PH domain 2 [Source:HGNC Symbol;Acc:HGNC:2721]
ENSG00000116983	HPCAL4	2,993568	hippocalcin like 4 [Source:HGNC Symbol;Acc:HGNC:18212]
ENSG00000152495	CAMK4	2,986456	calcium/calmodulin dependent protein kinase IV [Source:HGNC Symbol;Acc:HGNC:1464]
ENSG00000134313	KIDINS220	2,981868	kinase D interacting substrate 220 [Source:HGNC Symbol;Acc:HGNC:29508]
ENSG00000156486	KCNS2	2,968574	potassium voltage-gated channel modifier subfamily S member 2 [Source:HGNC Symbol;Acc:HGNC:6301]
ENSG00000166352	C11orf74	-2,96205	chromosome 11 open reading frame 74 [Source:HGNC Symbol;Acc:HGNC:25142]
ENSG00000106991	ENG	2,956984	endoglin [Source:HGNC Symbol;Acc:HGNC:3349]
ENSG00000116396	KCNC4	2,94063	potassium voltage-gated channel subfamily C member 4 [Source:HGNC Symbol;Acc:HGNC:6236]
ENSG00000176009	ASCL3	2,938682	achaete-scute family bHLH transcription factor 3 [Source:HGNC Symbol;Acc:HGNC:740]

**Biological theme:**	**Immune response**
**Gene sets:**	Activation of immune response (GO)
	Enlarged lymph nodes (MP)
	TCR signaling (Reactome)
	Downstream TCR signaling (Reactome)

**Ensembl gene ID**	**Gene**	**Weighted mean *z*-score**	**Description**
ENSG00000179344	HLA-DQB1	8,794715	major histocompatibility complex, class II, DQ beta 1 [Source:HGNC Symbol;Acc:HGNC:4944]
ENSG00000231389	HLA-DPA1	8,367733	major histocompatibility complex, class II, DP alpha 1 [Source:HGNC Symbol;Acc:HGNC:4938]
ENSG00000223865	HLA-DPB1	8,088377	major histocompatibility complex, class II, DP beta 1 [Source:HGNC Symbol;Acc:HGNC:4940]
ENSG00000229391	HLA-DRB6	7,754339	major histocompatibility complex, class II, DR beta 6 (pseudogene) [Source:HGNC Symbol;Acc:HGNC:4954]
ENSG00000019582	CD74	7,734311	CD74 molecule [Source:HGNC Symbol;Acc:HGNC:1697]
ENSG00000196735	HLA-DQA1	7,716375	major histocompatibility complex, class II, DQ alpha 1 [Source:HGNC Symbol;Acc:HGNC:4942]
ENSG00000204257	HLA-DMA	7,385088	major histocompatibility complex, class II, DM alpha [Source:HGNC Symbol;Acc:HGNC:4934]
ENSG00000242574	HLA-DMB	7,376177	major histocompatibility complex, class II, DM beta [Source:HGNC Symbol;Acc:HGNC:4935]
ENSG00000204287	HLA-DRA	7,251499	major histocompatibility complex, class II, DR alpha [Source:HGNC Symbol;Acc:HGNC:4947]
ENSG00000196126	HLA-DRB1	7,169586	major histocompatibility complex, class II, DR beta 1 [Source:HGNC Symbol;Acc:HGNC:4948]
ENSG00000179583	CIITA	6,56806	class II major histocompatibility complex transactivator [Source:HGNC Symbol;Acc:HGNC:7067]
ENSG00000204252	HLA-DOA	6,470649	major histocompatibility complex, class II, DO alpha [Source:HGNC Symbol;Acc:HGNC:4936]
ENSG00000232629	HLA-DQB2	5,587166	major histocompatibility complex, class II, DQ beta 2 [Source:HGNC Symbol;Acc:HGNC:4945]
ENSG00000211772	TRBC2	5,53476	T-cell receptor beta constant 2 [Source:HGNC Symbol;Acc:HGNC:12157]
ENSG00000182866	LCK	5,16429	LCK proto-oncogene, Src family tyrosine kinase [Source:HGNC Symbol;Acc:HGNC:6524]
ENSG00000116824	CD2	5,151253	CD2 molecule [Source:HGNC Symbol;Acc:HGNC:1639]
ENSG00000110448	CD5	5,100062	CD5 molecule [Source:HGNC Symbol;Acc:HGNC:1685]
ENSG00000227507	LTB	5,008763	lymphotoxin beta [Source:HGNC Symbol;Acc:HGNC:6711]
ENSG00000167286	CD3D	4,975573	CD3d molecule [Source:HGNC Symbol;Acc:HGNC:1673]
ENSG00000169442	CD52	4,941539	CD52 molecule [Source:HGNC Symbol;Acc:HGNC:1804]
ENSG00000013725	CD6	4,939083	CD6 molecule [Source:HGNC Symbol;Acc:HGNC:1691]
ENSG00000075884	ARHGAP15	4,908268	Rho GTPase activating protein 15 [Source:HGNC Symbol;Acc:HGNC:21030]
ENSG00000198821	CD247	4,863078	CD247 molecule [Source:HGNC Symbol;Acc:HGNC:1677]
ENSG00000147168	IL2RG	4,849608	interleukin 2 receptor subunit gamma [Source:HGNC Symbol;Acc:HGNC:6010]
ENSG00000198851	CD3E	4,811588	CD3e molecule [Source:HGNC Symbol;Acc:HGNC:1674]
ENSG00000009790	TRAF3IP3	4,809586	TRAF3 interacting protein 3 [Source:HGNC Symbol;Acc:HGNC:30766]
ENSG00000180096	SEPT1	4,774373	septin 1 [Source:HGNC Symbol;Acc:HGNC:2879]
ENSG00000110077	MS4A6A	4,691504	membrane spanning 4-domains A6A [Source:HGNC Symbol;Acc:HGNC:13375]
ENSG00000229164	NA	4,683413	NA
ENSG00000137078	SIT1	4,66575	signaling threshold regulating transmembrane adaptor 1 [Source:HGNC Symbol;Acc:HGNC:17710]

ENSG00000115085	ZAP70	4,655719	zeta chain of T-cell receptor associated protein kinase 70 [Source:HGNC Symbol;Acc:HGNC:12858]
ENSG00000010610	CD4	4,612142	CD4 molecule [Source:HGNC Symbol;Acc:HGNC:1678]
ENSG00000113263	ITK	4,601818	IL2 inducible T-cell kinase [Source:HGNC Symbol;Acc:HGNC:6171]
ENSG00000081237	PTPRC	4,572809	protein tyrosine phosphatase, receptor type C [Source:HGNC Symbol;Acc:HGNC:9666]
ENSG00000180353	HCLS1	4,548955	hematopoietic cell-specific Lyn substrate 1 [Source:HGNC Symbol;Acc:HGNC:4844]
ENSG00000224557	HLA-DPB2	4,518237	major histocompatibility complex, class II, DP beta 2 (pseudogene) [Source:HGNC Symbol;Acc:HGNC:4941]
ENSG00000160654	CD3G	4,470402	CD3g molecule [Source:HGNC Symbol;Acc:HGNC:1675]
ENSG00000161570	NA	4,454095	NA
ENSG00000089012	SIRPG	4,427965	signal regulatory protein gamma [Source:HGNC Symbol;Acc:HGNC:15757]
ENSG00000143119	CD53	4,424963	CD53 molecule [Source:HGNC Symbol;Acc:HGNC:1686]
ENSG00000141293	SKAP1	4,419774	src kinase associated phosphoprotein 1 [Source:HGNC Symbol;Acc:HGNC:15605]
ENSG00000043462	LCP2	4,410254	lymphocyte cytosolic protein 2 [Source:HGNC Symbol;Acc:HGNC:6529]
ENSG00000104894	CD37	4,379604	CD37 molecule [Source:HGNC Symbol;Acc:HGNC:1666]
ENSG00000082074	FYB	4,375921	FYN binding protein [Source:HGNC Symbol;Acc:HGNC:4036]
ENSG00000185862	EVI2B	4,364709	ecotropic viral integration site 2B [Source:HGNC Symbol;Acc:HGNC:3500]
ENSG00000153283	CD96	4,343347	CD96 molecule [Source:HGNC Symbol;Acc:HGNC:16892]
ENSG00000111796	KLRB1	4,330489	killer cell lectin like receptor B1 [Source:HGNC Symbol;Acc:HGNC:6373]
ENSG00000110031	LPXN	4,32642	leupaxin [Source:HGNC Symbol;Acc:HGNC:14061]
ENSG00000126264	HCST	4,319963	hematopoietic cell signal transducer [Source:HGNC Symbol;Acc:HGNC:16977]
ENSG00000100385	IL2RB	4,314786	interleukin 2 receptor subunit beta [Source:HGNC Symbol;Acc:HGNC:6009]
ENSG00000105122	RASAL3	4,267707	RAS protein activator like 3 [Source:HGNC Symbol;Acc:HGNC:26129]
ENSG00000160185	UBASH3A	4,266742	ubiquitin associated and SH3 domain containing A [Source:HGNC Symbol;Acc:HGNC:12462]
ENSG00000138755	CXCL9	4,233919	C-X-C motif chemokine ligand 9 [Source:HGNC Symbol;Acc:HGNC:7098]
ENSG00000163519	TRAT1	4,207095	T-cell receptor associated transmembrane adaptor 1 [Source:HGNC Symbol;Acc:HGNC:30698]
ENSG00000123329	ARHGAP9	4,204577	Rho GTPase activating protein 9 [Source:HGNC Symbol;Acc:HGNC:14130]
ENSG00000162894	FCMR	4,196386	Fc fragment of IgM receptor [Source:HGNC Symbol;Acc:HGNC:14315]
ENSG00000127152	BCL11B	4,194725	B-cell CLL/lymphoma 11B [Source:HGNC Symbol;Acc:HGNC:13222]
ENSG00000145649	GZMA	4,191488	granzyme A [Source:HGNC Symbol;Acc:HGNC:4708]
ENSG00000186265	BTLA	4,190389	B and T lymphocyte associated [Source:HGNC Symbol;Acc:HGNC:21087]
ENSG00000183918	SH2D1A	4,186017	SH2 domain containing 1A [Source:HGNC Symbol;Acc:HGNC:10820]
ENSG00000162511	LAPTM5	4,119971	lysosomal protein transmembrane 5 [Source:HGNC Symbol;Acc:HGNC:29612]
ENSG00000179934	CCR8	4,118955	C-C motif chemokine receptor 8 [Source:HGNC Symbol;Acc:HGNC:1609]
ENSG00000134516	DOCK2	4,111048	dedicator of cytokinesis 2 [Source:HGNC Symbol;Acc:HGNC:2988]
ENSG00000112799	LY86	4,110642	lymphocyte antigen 86 [Source:HGNC Symbol;Acc:HGNC:16837]
ENSG00000175463	TBC1D10C	4,091699	TBC1 domain family member 10C [Source:HGNC Symbol;Acc:HGNC:24702]
ENSG00000110848	CD69	4,075173	CD69 molecule [Source:HGNC Symbol;Acc:HGNC:1694]
ENSG00000231461	HLA-DPA2	4,069831	major histocompatibility complex, class II, DP alpha 2 (pseudogene) [Source:HGNC Symbol;Acc:HGNC:4939]

ENSG00000174946	GPR171	4,0554	G protein-coupled receptor 171 [Source:HGNC Symbol;Acc:HGNC:30057]
ENSG00000213402	PTPRCAP	4,051938	protein tyrosine phosphatase, receptor type C associated protein [Source:HGNC Symbol;Acc:HGNC:9667]
ENSG00000134242	PTPN22	4,050617	protein tyrosine phosphatase, non-receptor type 22 [Source:HGNC Symbol;Acc:HGNC:9652]
ENSG00000143390	RFX5	4,037235	regulatory factor X5 [Source:HGNC Symbol;Acc:HGNC:9986]
ENSG00000104814	MAP4K1	4,037143	mitogen-activated protein kinase kinase kinase kinase 1 [Source:HGNC Symbol;Acc:HGNC:6863]
ENSG00000169413	RNASE6	4,034963	ribonuclease A family member k6 [Source:HGNC Symbol;Acc:HGNC:10048]
ENSG00000173762	CD7	4,034455	CD7 molecule [Source:HGNC Symbol;Acc:HGNC:1695]
ENSG00000211899	IGHM	4,032006	immunoglobulin heavy constant mu [Source:HGNC Symbol;Acc:HGNC:5541]
ENSG00000168421	RHOH	4,031815	ras homolog family member H [Source:HGNC Symbol;Acc:HGNC:686]
ENSG00000148908	RGS10	4,002662	regulator of G protein signaling 10 [Source:HGNC Symbol;Acc:HGNC:9992]
ENSG00000112149	CD83	4,000381	CD83 molecule [Source:HGNC Symbol;Acc:HGNC:1703]
ENSG00000126353	CCR7	3,976686	C-C motif chemokine receptor 7 [Source:HGNC Symbol;Acc:HGNC:1608]
ENSG00000162739	SLAMF6	3,967657	SLAM family member 6 [Source:HGNC Symbol;Acc:HGNC:21392]
ENSG00000168685	IL7R	3,963672	interleukin 7 receptor [Source:HGNC Symbol;Acc:HGNC:6024]
ENSG00000167984	NLRC3	3,941012	NLR family CARD domain containing 3 [Source:HGNC Symbol;Acc:HGNC:29889]
ENSG00000188820	FAM26F	3,938207	family with sequence similarity 26 member F [Source:HGNC Symbol;Acc:HGNC:33391]
ENSG00000211785	TRAV12-1	3,906063	T-cell receptor alpha variable 12-1 [Source:HGNC Symbol;Acc:HGNC:12105]
ENSG00000078589	P2RY10	3,89986	purinergic receptor P2Y10 [Source:HGNC Symbol;Acc:HGNC:19906]
ENSG00000186517	ARHGAP30	3,898922	Rho GTPase activating protein 30 [Source:HGNC Symbol;Acc:HGNC:27414]
ENSG00000102879	CORO1A	3,891896	coronin 1A [Source:HGNC Symbol;Acc:HGNC:2252]
ENSG00000213658	LAT	3,886074	linker for activation of T-cells [Source:HGNC Symbol;Acc:HGNC:18874]
ENSG00000163600	ICOS	3,883388	inducible T-cell costimulator [Source:HGNC Symbol;Acc:HGNC:5351]
ENSG00000115165	CYTIP	3,882563	cytohesin 1 interacting protein [Source:HGNC Symbol;Acc:HGNC:9506]
ENSG00000136167	LCP1	3,880266	lymphocyte cytosolic protein 1 [Source:HGNC Symbol;Acc:HGNC:6528]
ENSG00000108798	ABI3	3,858397	ABI family member 3 [Source:HGNC Symbol;Acc:HGNC:29859]
ENSG00000072818	ACAP1	3,851821	ArfGAP with coiled-coil, ankyrin repeat and PH domains 1 [Source:HGNC Symbol;Acc:HGNC:16467]
ENSG00000153563	CD8A	3,81902	CD8a molecule [Source:HGNC Symbol;Acc:HGNC:1706]
ENSG00000005844	ITGAL	3,803476	integrin subunit alpha L [Source:HGNC Symbol;Acc:HGNC:6148]
ENSG00000008517	IL32	3,797842	interleukin 32 [Source:HGNC Symbol;Acc:HGNC:16830]
ENSG00000179144	GIMAP7	3,787008	GTPase, IMAP family member 7 [Source:HGNC Symbol;Acc:HGNC:22404]
ENSG00000172575	RASGRP1	3,784238	RAS guanyl releasing protein 1 [Source:HGNC Symbol;Acc:HGNC:9878]
ENSG00000180448	ARHGAP45	3,771771	Rho GTPase activating protein 45 [Source:HGNC Symbol;Acc:HGNC:17102]
ENSG00000172543	CTSW	3,748069	cathepsin W [Source:HGNC Symbol;Acc:HGNC:2546]

**Biological theme:**	**Caspase cascade/apoptosis**
**Gene sets:**	NOD1 subnetwork (ENSG)

**Ensembl gene ID**	**Gene**	**Weighted mean *z*-score**	**Description**
ENSG00000132002	DNAJB1	8,625188	DnaJ heat shock protein family (Hsp40) member B1 [Source:HGNC Symbol;Acc:HGNC:5270]
ENSG00000204388	HSPA1B	8,296803	heat shock protein family A (Hsp70) member 1B [Source:HGNC Symbol;Acc:HGNC:5233]
ENSG00000144381	HSPD1	7,757833	heat shock protein family D (Hsp60) member 1 [Source:HGNC Symbol;Acc:HGNC:5261]
ENSG00000204390	HSPA1L	7,514735	heat shock protein family A (Hsp70) member 1 like [Source:HGNC Symbol;Acc:HGNC:5234]
ENSG00000120694	HSPH1	7,037116	heat shock protein family H (Hsp110) member 1 [Source:HGNC Symbol;Acc:HGNC:16969]
ENSG00000204389	HSPA1A	7,033475	heat shock protein family A (Hsp70) member 1A [Source:HGNC Symbol;Acc:HGNC:5232]
ENSG00000086061	DNAJA1	6,953057	DnaJ heat shock protein family (Hsp40) member A1 [Source:HGNC Symbol;Acc:HGNC:5229]
ENSG00000140403	DNAJA4	5,928391	DnaJ heat shock protein family (Hsp40) member A4 [Source:HGNC Symbol;Acc:HGNC:14885]
ENSG00000162616	DNAJB4	5,817007	DnaJ heat shock protein family (Hsp40) member B4 [Source:HGNC Symbol;Acc:HGNC:14886]
ENSG00000168404	MLKL	5,669237	mixed lineage kinase domain like pseudokinase [Source:HGNC Symbol;Acc:HGNC:26617]
ENSG00000159873	CCDC117	5,634329	coiled-coil domain containing 117 [Source:HGNC Symbol;Acc:HGNC:26599]
ENSG00000196954	CASP4	5,505875	caspase 4 [Source:HGNC Symbol;Acc:HGNC:1505]
ENSG00000117226	GBP3	5,465861	guanylate binding protein 3 [Source:HGNC Symbol;Acc:HGNC:4184]
ENSG00000100591	AHSA1	5,167707	activator of HSP90 ATPase activity 1 [Source:HGNC Symbol;Acc:HGNC:1189]
ENSG00000105939	ZC3HAV1	5,077428	zinc finger CCCH-type containing, antiviral 1 [Source:HGNC Symbol;Acc:HGNC:23721]
ENSG00000160570	DEDD2	5,065977	death effector domain containing 2 [Source:HGNC Symbol;Acc:HGNC:24450]
ENSG00000235505		5,059363	
ENSG00000110172	CHORDC1	4,913954	cysteine and histidine rich domain containing 1 [Source:HGNC Symbol;Acc:HGNC:14525]
ENSG00000116161	CACYBP	4,76213	calcyclin binding protein [Source:HGNC Symbol;Acc:HGNC:30423]
ENSG00000003400	CASP10	4,612144	caspase 10 [Source:HGNC Symbol;Acc:HGNC:1500]
ENSG00000080824	HSP90AA1	4,584465	heat shock protein 90 alpha family class A member 1 [Source:HGNC Symbol;Acc:HGNC:5253]
ENSG00000173209	AHSA2	4,557669	activator of HSP90 ATPase homolog 2 [Source:HGNC Symbol;Acc:HGNC:20437]
ENSG00000204397	CARD16	4,518489	caspase recruitment domain family member 16 [Source:HGNC Symbol;Acc:HGNC:33701]
ENSG00000151929	BAG3	4,404858	BCL2 associated athanogene 3 [Source:HGNC Symbol;Acc:HGNC:939]
ENSG00000132952	USPL1	4,347362	ubiquitin specific peptidase like 1 [Source:HGNC Symbol;Acc:HGNC:20294]
ENSG00000004478	FKBP4	4,330958	FK506 binding protein 4 [Source:HGNC Symbol;Acc:HGNC:3720]
ENSG00000112110	MRPL18	4,313322	mitochondrial ribosomal protein L18 [Source:HGNC Symbol;Acc:HGNC:14477]
ENSG00000115541	HSPE1	4,311942	heat shock protein family E (Hsp10) member 1 [Source:HGNC Symbol;Acc:HGNC:5269]
ENSG00000182359	KBTBD3	4,107999	kelch repeat and BTB domain containing 3 [Source:HGNC Symbol;Acc:HGNC:22934]
ENSG00000182827	ACBD3	4,068569	acyl-CoA binding domain containing 3 [Source:HGNC Symbol;Acc:HGNC:15453]
ENSG00000234127	TRIM26	4,026903	tripartite motif containing 26 [Source:HGNC Symbol;Acc:HGNC:12962]
ENSG00000112343	TRIM38	4,017224	tripartite motif containing 38 [Source:HGNC Symbol;Acc:HGNC:10059]
ENSG00000204403	CASP12	4,009687	caspase 12 (gene/pseudogene) [Source:HGNC Symbol;Acc:HGNC:19004]

ENSG00000188917	TRMT2B	3,971682	tRNA methyltransferase 2 homolog B [Source:HGNC Symbol;Acc:HGNC:25748]
ENSG00000117222	RBBP5	3,912675	RB binding protein 5, histone lysine methyltransferase complex subunit [Source:HGNC Symbol;Acc:HGNC:9888]
ENSG00000184378	ACTRT3	3,891801	actin related protein T3 [Source:HGNC Symbol;Acc:HGNC:24022]
ENSG00000181481	RNF135	3,861298	ring finger protein 135 [Source:HGNC Symbol;Acc:HGNC:21158]
ENSG00000125347	IRF1	3,858315	interferon regulatory factor 1 [Source:HGNC Symbol;Acc:HGNC:6116]
ENSG00000064012	CASP8	3,853201	caspase 8 [Source:HGNC Symbol;Acc:HGNC:1509]
ENSG00000178381	ZFAND2A	3,819473	zinc finger AN1-type containing 2A [Source:HGNC Symbol;Acc:HGNC:28073]
ENSG00000096654	ZNF184	3,816668	zinc finger protein 184 [Source:HGNC Symbol;Acc:HGNC:12975]
ENSG00000137757	CASP5	3,788259	caspase 5 [Source:HGNC Symbol;Acc:HGNC:1506]
ENSG00000121858	TNFSF10	3,787032	TNF superfamily member 10 [Source:HGNC Symbol;Acc:HGNC:11925]
ENSG00000197714	ZNF460	3,767548	zinc finger protein 460 [Source:HGNC Symbol;Acc:HGNC:21628]
ENSG00000249281	NA	3,748099	NA
ENSG00000110852	CLEC2B	3,712507	C-type lectin domain family 2 member B [Source:HGNC Symbol;Acc:HGNC:2053]
ENSG00000013441	CLK1	3,670167	CDC like kinase 1 [Source:HGNC Symbol;Acc:HGNC:2068]
ENSG00000165806	CASP7	3,669723	caspase 7 [Source:HGNC Symbol;Acc:HGNC:1508]
ENSG00000174130	TLR6	3,658076	toll like receptor 6 [Source:HGNC Symbol;Acc:HGNC:16711]
ENSG00000006625	GGCT	3,649888	gamma-glutamylcyclotransferase [Source:HGNC Symbol;Acc:HGNC:21705]
ENSG00000183323	CCDC125	3,648215	coiled-coil domain containing 125 [Source:HGNC Symbol;Acc:HGNC:28924]
ENSG00000206418	RAB12	3,642415	RAB12, member RAS oncogene family [Source:HGNC Symbol;Acc:HGNC:31332]
ENSG00000137752	CASP1	3,628547	caspase 1 [Source:HGNC Symbol;Acc:HGNC:1499]
ENSG00000144401	METTL21A	3,617536	methyltransferase like 21A [Source:HGNC Symbol;Acc:HGNC:30476]
ENSG00000106211	HSPB1	3,592471	heat shock protein family B (small) member 1 [Source:HGNC Symbol;Acc:HGNC:5246]
ENSG00000230037	UBBP1	3,591736	ubiquitin B pseudogene 1 [Source:HGNC Symbol;Acc:HGNC:12464]
ENSG00000237298	TTN-AS1	3,587822	TTN antisense RNA 1 [Source:HGNC Symbol;Acc:HGNC:44124]
ENSG00000168405	CMAHP	3,584939	cytidine monophospho-N-acetylneuraminic acid hydroxylase, pseudogene [Source:HGNC Symbol;Acc:HGNC:2098]
ENSG00000110330	BIRC2	3,551986	baculoviral IAP repeat containing 2 [Source:HGNC Symbol;Acc:HGNC:590]
ENSG00000225946	NA	-3,5333	NA
ENSG00000147852	VLDLR	-3,53328	very low density lipoprotein receptor [Source:HGNC Symbol;Acc:HGNC:12698]
ENSG00000117616	RSRP1	3,522937	arginine and serine rich protein 1 [Source:HGNC Symbol;Acc:HGNC:25234]
ENSG00000172530	BANP	3,511046	BTG3 associated nuclear protein [Source:HGNC Symbol;Acc:HGNC:13450]
ENSG00000164308	ERAP2	3,50351	endoplasmic reticulum aminopeptidase 2 [Source:HGNC Symbol;Acc:HGNC:29499]
ENSG00000201340		-3,49244	Y RNA [Source:RFAM;Acc:RF00019]
ENSG00000172738	TMEM217	3,481207	transmembrane protein 217 [Source:HGNC Symbol;Acc:HGNC:21238]
ENSG00000164430	MB21D1	3,472955	Mab-21 domain containing 1 [Source:HGNC Symbol;Acc:HGNC:21367]
ENSG00000104320	NBN	3,471579	nibrin [Source:HGNC Symbol;Acc:HGNC:7652]
ENSG00000007129	CEACAM21	3,456471	carcinoembryonic antigen related cell adhesion molecule 21 [Source:HGNC Symbol;Acc:HGNC:28834]
ENSG00000197442	MAP3K5	3,454716	mitogen-activated protein kinase kinase kinase 5 [Source:HGNC Symbol;Acc:HGNC:6857]

ENSG00000219807	ARF1P1	3,450048	ADP ribosylation factor 1 pseudogene 1 [Source:HGNC Symbol;Acc:HGNC:22500]
ENSG00000136560	TANK	3,429337	TRAF family member associated NFKB activator [Source:HGNC Symbol;Acc:HGNC:11562]
ENSG00000141569	TRIM65	3,427981	tripartite motif containing 65 [Source:HGNC Symbol;Acc:HGNC:27316]
ENSG00000111371	SLC38A1	-3,42479	solute carrier family 38 member 1 [Source:HGNC Symbol;Acc:HGNC:13447]
ENSG00000145365	TIFA	3,418926	TRAF interacting protein with forkhead associated domain [Source:HGNC Symbol;Acc:HGNC:19075]
ENSG00000167333	TRIM68	3,406361	tripartite motif containing 68 [Source:HGNC Symbol;Acc:HGNC:21161]
ENSG00000132680	KIAA0907	3,390356	KIAA0907 [Source:HGNC Symbol;Acc:HGNC:29145]
ENSG00000105854	PON2	3,366168	paraoxonase 2 [Source:HGNC Symbol;Acc:HGNC:9205]
ENSG00000187116	LILRA5	3,36525	leukocyte immunoglobulin like receptor A5 [Source:HGNC Symbol;Acc:HGNC:16309]
ENSG00000170903	MSANTD4	3,362203	Myb/SANT DNA binding domain containing 4 with coiled-coils [Source:HGNC Symbol;Acc:HGNC:29383]
ENSG00000026950	BTN3A1	3,330896	butyrophilin subfamily 3 member A1 [Source:HGNC Symbol;Acc:HGNC:1138]
ENSG00000189060	H1F0	-3,32882	H1 histone family member 0 [Source:HGNC Symbol;Acc:HGNC:4714]
ENSG00000131979	GCH1	3,324423	GTP cyclohydrolase 1 [Source:HGNC Symbol;Acc:HGNC:4193]
ENSG00000123636	BAZ2B	3,322775	bromodomain adjacent to zinc finger domain 2B [Source:HGNC Symbol;Acc:HGNC:963]
ENSG00000172716	SLFN11	3,300881	schlafen family member 11 [Source:HGNC Symbol;Acc:HGNC:26633]
ENSG00000232874		-3,27779	
ENSG00000164691	TAGAP	3,271943	T-cell activation RhoGTPase activating protein [Source:HGNC Symbol;Acc:HGNC:15669]
ENSG00000247624	CPEB2-AS1	3,269631	CPEB2 antisense RNA 1 (head to head) [Source:HGNC Symbol;Acc:HGNC:49082]
ENSG00000162645	GBP2	3,26542	guanylate binding protein 2 [Source:HGNC Symbol;Acc:HGNC:4183]
ENSG00000140563	MCTP2	3,263248	multiple C2 and transmembrane domain containing 2 [Source:HGNC Symbol;Acc:HGNC:25636]
ENSG00000168439	STIP1	3,233948	stress induced phosphoprotein 1 [Source:HGNC Symbol;Acc:HGNC:11387]
ENSG00000154589	LY96	3,213273	lymphocyte antigen 96 [Source:HGNC Symbol;Acc:HGNC:17156]
ENSG00000158079	PTPDC1	-3,2022	protein tyrosine phosphatase domain containing 1 [Source:HGNC Symbol;Acc:HGNC:30184]
ENSG00000164729	SLC35G3	-3,19783	solute carrier family 35 member G3 [Source:HGNC Symbol;Acc:HGNC:26848]
ENSG00000228247	UBBP2	3,197343	ubiquitin B pseudogene 2 [Source:HGNC Symbol;Acc:HGNC:12465]
ENSG00000130270	ATP8B3	-3,19065	ATPase phospholipid transporting 8B3 [Source:HGNC Symbol;Acc:HGNC:13535]
ENSG00000118260	CREB1	3,188116	cAMP responsive element binding protein 1 [Source:HGNC Symbol;Acc:HGNC:2345]
ENSG00000128284	APOL3	3,183511	apolipoprotein L3 [Source:HGNC Symbol;Acc:HGNC:14868]
ENSG00000215458	AATBC	3,180381	apoptosis associated transcript in bladder cancer [Source:HGNC Symbol;Acc:HGNC:51526]
ENSG00000149231	CCDC82	3,180336	coiled-coil domain containing 82 [Source:HGNC Symbol;Acc:HGNC:26282]

**Biological theme:**	**Phosphatase activity**
**Gene sets:**	Phosphatase activity (GO)
	Phosphoprotein phosphatase activity (GO)
	Phosphoric ester hydrolase activity (GO)

Ensembl gene ID	Gene	Weighted mean *z* -score	Description
ENSG00000176407	KCMF1	3,864457	potassium channel modulatory factor 1 [Source:HGNC Symbol;Acc:HGNC:20589]
ENSG00000100532	CGRRF1	3,749549	cell growth regulator with ring finger domain 1 [Source:HGNC Symbol;Acc:HGNC:15528]
ENSG00000073417	PDE8A	3,608651	phosphodiesterase 8A [Source:HGNC Symbol;Acc:HGNC:8793]
ENSG00000135932	CAB39	3,590708	calcium binding protein 39 [Source:HGNC Symbol;Acc:HGNC:20292]
ENSG00000250544	LINC02059	3,46055	long intergenic non-protein coding RNA 2059 [Source:HGNC Symbol;Acc:HGNC:52902]
ENSG00000008853	RHOBTB2	3,453581	Rho related BTB domain containing 2 [Source:HGNC Symbol;Acc:HGNC:18756]
ENSG00000138032	PPM1B	3,419349	protein phosphatase, Mg2+/Mn2+ dependent 1B [Source:HGNC Symbol;Acc:HGNC:9276]
ENSG00000176571	CNBD1	3,372357	cyclic nucleotide binding domain containing 1 [Source:HGNC Symbol;Acc:HGNC:26663]
ENSG00000008394	MGST1	-3,36091	microsomal glutathione S-transferase 1 [Source:HGNC Symbol;Acc:HGNC:7061]
ENSG00000204634	TBC1D8	3,300874	TBC1 domain family member 8 [Source:HGNC Symbol;Acc:HGNC:17791]
ENSG00000163788	SNRK	3,293715	SNF related kinase [Source:HGNC Symbol;Acc:HGNC:30598]
ENSG00000008441	NFIX	-3,28952	nuclear factor I X [Source:HGNC Symbol;Acc:HGNC:7788]
ENSG00000234859		-3,26935	
ENSG00000122042	UBL3	3,255509	ubiquitin like 3 [Source:HGNC Symbol;Acc:HGNC:12504]
ENSG00000223414	LINC00473	3,241392	long intergenic non-protein coding RNA 473 [Source:HGNC Symbol;Acc:HGNC:21160]
ENSG00000173230	GOLGB1	-3,23783	golgin B1 [Source:HGNC Symbol;Acc:HGNC:4429]
ENSG00000173041	ZNF680	-3,22329	zinc finger protein 680 [Source:HGNC Symbol;Acc:HGNC:26897]
ENSG00000132640	BTBD3	3,214181	BTB domain containing 3 [Source:HGNC Symbol;Acc:HGNC:15854]
ENSG00000132326	PER2	3,200003	period circadian clock 2 [Source:HGNC Symbol;Acc:HGNC:8846]
ENSG00000120910	PPP3CC	3,191223	protein phosphatase 3 catalytic subunit gamma [Source:HGNC Symbol;Acc:HGNC:9316]
ENSG00000135960	EDAR	-3,19091	ectodysplasin A receptor [Source:HGNC Symbol;Acc:HGNC:2895]
ENSG00000114302	PRKAR2A	3,174658	protein kinase cAMP-dependent type II regulatory subunit alpha [Source:HGNC Symbol;Acc:HGNC:9391]
ENSG00000107758	PPP3CB	3,164928	protein phosphatase 3 catalytic subunit beta [Source:HGNC Symbol;Acc:HGNC:9315]
ENSG00000198039	ZNF273	-3,12387	zinc finger protein 273 [Source:HGNC Symbol;Acc:HGNC:13067]
ENSG00000176273	SLC35G1	3,120439	solute carrier family 35 member G1 [Source:HGNC Symbol;Acc:HGNC:26607]
ENSG00000119986	AVPI1	3,119359	arginine vasopressin induced 1 [Source:HGNC Symbol;Acc:HGNC:30898]
ENSG00000065809	FAM107B	3,104941	family with sequence similarity 107 member B [Source:HGNC Symbol;Acc:HGNC:23726]
ENSG00000165914	TTC7B	3,096498	tetratricopeptide repeat domain 7B [Source:HGNC Symbol;Acc:HGNC:19858]
ENSG00000132359	RAP1GAP2	3,080589	RAP1 GTPase activating protein 2 [Source:HGNC Symbol;Acc:HGNC:29176]
ENSG00000198833	UBE2J1	3,071382	ubiquitin conjugating enzyme E2 J1 [Source:HGNC Symbol;Acc:HGNC:17598]
ENSG00000110514	MADD	3,062192	MAP kinase activating death domain [Source:HGNC Symbol;Acc:HGNC:6766]

ENSG00000108788	MLX	3,055069	MLX, MAX dimerization protein [Source:HGNC Symbol;Acc:HGNC:11645]
ENSG00000133731	IMPA1	3,041034	inositol monophosphatase 1 [Source:HGNC Symbol;Acc:HGNC:6050]
ENSG00000197442	MAP3K5	3,03696	mitogen-activated protein kinase kinase kinase 5 [Source:HGNC Symbol;Acc:HGNC:6857]
ENSG00000172361	CFAP53	-3,02168	cilia and flagella associated protein 53 [Source:HGNC Symbol;Acc:HGNC:26530]
ENSG00000106261	ZKSCAN1	-3,01616	zinc finger with KRAB and SCAN domains 1 [Source:HGNC Symbol;Acc:HGNC:13101]
ENSG00000040933	INPP4A	3,010244	inositol polyphosphate-4-phosphatase type I A [Source:HGNC Symbol;Acc:HGNC:6074]
ENSG00000070961	ATP2B1	2,988601	ATPase plasma membrane Ca2+ transporting 1 [Source:HGNC Symbol;Acc:HGNC:814]
ENSG00000110697	PITPNM1	2,972496	phosphatidylinositol transfer protein membrane associated 1 [Source:HGNC Symbol;Acc:HGNC:9003]
ENSG00000174839	DENND6A	2,967336	DENN domain containing 6A [Source:HGNC Symbol;Acc:HGNC:26635]
ENSG00000129158	SERGEF	2,961886	secretion regulating guanine nucleotide exchange factor [Source:HGNC Symbol;Acc:HGNC:17499]
ENSG00000198113	TOR4A	2,961361	torsin family 4 member A [Source:HGNC Symbol;Acc:HGNC:25981]
ENSG00000256546		-2,95933	
ENSG00000138698	RAP1GDS1	2,949026	Rap1 GTPase-GDP dissociation stimulator 1 [Source:HGNC Symbol;Acc:HGNC:9859]
ENSG00000179094	PER1	2,943918	period circadian clock 1 [Source:HGNC Symbol;Acc:HGNC:8845]
ENSG00000109756	RAPGEF2	2,935232	Rap guanine nucleotide exchange factor 2 [Source:HGNC Symbol;Acc:HGNC:16854]
ENSG00000162852	CNST	2,934987	consortin, connexin sorting protein [Source:HGNC Symbol;Acc:HGNC:26486]
ENSG00000130511	SSBP4	2,930757	single stranded DNA binding protein 4 [Source:HGNC Symbol;Acc:HGNC:15676]
ENSG00000027075	PRKCH	2,918549	protein kinase C eta [Source:HGNC Symbol;Acc:HGNC:9403]
ENSG00000139289	PHLDA1	2,912259	pleckstrin homology like domain family A member 1 [Source:HGNC Symbol;Acc:HGNC:8933]
ENSG00000114166	KAT2B	2,904815	lysine acetyltransferase 2B [Source:HGNC Symbol;Acc:HGNC:8638]
ENSG00000258776		-2,90312	
ENSG00000175832	ETV4	2,902654	ETS variant 4 [Source:HGNC Symbol;Acc:HGNC:3493]
ENSG00000118276	B4GALT6	2,898231	beta-1,4-galactosyltransferase 6 [Source:HGNC Symbol;Acc:HGNC:929]
ENSG00000164621	SMAD5-AS1	-2,89657	SMAD5 antisense RNA 1 [Source:HGNC Symbol;Acc:HGNC:30586]
ENSG00000244405	ETV5	2,887049	ETS variant 5 [Source:HGNC Symbol;Acc:HGNC:3494]
ENSG00000187678	SPRY4	2,886788	sprouty RTK signaling antagonist 4 [Source:HGNC Symbol;Acc:HGNC:15533]
ENSG00000213047	DENND1B	2,878624	DENN domain containing 1B [Source:HGNC Symbol;Acc:HGNC:28404]
ENSG00000197826	C4orf22	-2,874	chromosome 4 open reading frame 22 [Source:HGNC Symbol;Acc:HGNC:28554]
ENSG00000145743	FBXL17	2,860239	F-box and leucine rich repeat protein 17 [Source:HGNC Symbol;Acc:HGNC:13615]
ENSG00000143952	VPS54	2,853395	VPS54, GARP complex subunit [Source:HGNC Symbol;Acc:HGNC:18652]
ENSG00000138101	DTNB	2,842534	dystrobrevin beta [Source:HGNC Symbol;Acc:HGNC:3058]
ENSG00000213639	PPP1CB	2,838167	protein phosphatase 1 catalytic subunit beta [Source:HGNC Symbol;Acc:HGNC:9282]
ENSG00000124151	NCOA3	2,834192	nuclear receptor coactivator 3 [Source:HGNC Symbol;Acc:HGNC:7670]
ENSG00000182224	CYB5D1	-2,83028	cytochrome b5 domain containing 1 [Source:HGNC Symbol;Acc:HGNC:26516]
ENSG00000225539	LINC01821	-2,82904	long intergenic non-protein coding RNA 1821 [Source:HGNC Symbol;Acc:HGNC:52626]
ENSG00000160959	LRRC14	-2,82544	leucine rich repeat containing 14 [Source:HGNC Symbol;Acc:HGNC:20419]
ENSG00000069956	MAPK6	2,82137	mitogen-activated protein kinase 6 [Source:HGNC Symbol;Acc:HGNC:6879]

ENSG00000236432		-2,82008	
ENSG00000171804	WDR87	-2,81123	WD repeat domain 87 [Source:HGNC Symbol;Acc:HGNC:29934]
ENSG00000132016	C19orf57	-2,81073	chromosome 19 open reading frame 57 [Source:HGNC Symbol;Acc:HGNC:28153]
ENSG00000065802	ASB1	2,808504	ankyrin repeat and SOCS box containing 1 [Source:HGNC Symbol;Acc:HGNC:16011]
ENSG00000156875	MFSD14A	2,802228	major facilitator superfamily domain containing 14A [Source:HGNC Symbol;Acc:HGNC:23363]
ENSG00000171530	TBCA	-2,79611	tubulin folding cofactor A [Source:HGNC Symbol;Acc:HGNC:11579]
ENSG00000182500	NA	2,795892	NA
ENSG00000135002	RFK	2,79467	riboflavin kinase [Source:HGNC Symbol;Acc:HGNC:30324]
ENSG00000196247	ZNF107	-2,78833	zinc finger protein 107 [Source:HGNC Symbol;Acc:HGNC:12887]
ENSG00000174574	AKIRIN1	2,787902	akirin 1 [Source:HGNC Symbol;Acc:HGNC:25744]
ENSG00000100605	ITPK1	2,785637	inositol-tetrakisphosphate 1-kinase [Source:HGNC Symbol;Acc:HGNC:6177]
ENSG00000196865	NHLRC2	2,785384	NHL repeat containing 2 [Source:HGNC Symbol;Acc:HGNC:24731]
ENSG00000198106		-2,78275	
ENSG00000198668	CALM1	2,775872	calmodulin 1 [Source:HGNC Symbol;Acc:HGNC:1442]
ENSG00000169946	ZFPM2	-2,77344	zinc finger protein, FOG family member 2 [Source:HGNC Symbol;Acc:HGNC:16700]
ENSG00000158113	LRRC43	-2,76577	leucine rich repeat containing 43 [Source:HGNC Symbol;Acc:HGNC:28562]
ENSG00000173334	TRIB1	2,763587	tribbles pseudokinase 1 [Source:HGNC Symbol;Acc:HGNC:16891]
ENSG00000175387	SMAD2	2,757901	SMAD family member 2 [Source:HGNC Symbol;Acc:HGNC:6768]
ENSG00000107854	TNKS2	2,753637	tankyrase 2 [Source:HGNC Symbol;Acc:HGNC:15677]
ENSG00000164253	WDR41	2,75192	WD repeat domain 41 [Source:HGNC Symbol;Acc:HGNC:25601]
ENSG00000153310	FAM49B	2,748118	family with sequence similarity 49 member B [Source:HGNC Symbol;Acc:HGNC:25216]
ENSG00000010810	FYN	2,740599	FYN proto-oncogene, Src family tyrosine kinase [Source:HGNC Symbol;Acc:HGNC:4037]
ENSG00000106789	CORO2A	2,737482	coronin 2A [Source:HGNC Symbol;Acc:HGNC:2255]
ENSG00000154822	PLCL2	2,735062	phospholipase C like 2 [Source:HGNC Symbol;Acc:HGNC:9064]
ENSG00000159346	ADIPOR1	2,72892	adiponectin receptor 1 [Source:HGNC Symbol;Acc:HGNC:24040]
ENSG00000165355	FBXO33	2,726795	F-box protein 33 [Source:HGNC Symbol;Acc:HGNC:19833]
ENSG00000038427	VCAN	-2,72399	versican [Source:HGNC Symbol;Acc:HGNC:2464]
ENSG00000138134	STAMBPL1	2,716632	STAM binding protein like 1 [Source:HGNC Symbol;Acc:HGNC:24105]
ENSG00000109466	KLHL2	2,712344	kelch like family member 2 [Source:HGNC Symbol;Acc:HGNC:6353]
ENSG00000106803	SEC61B	-2,71048	Sec61 translocon beta subunit [Source:HGNC Symbol;Acc:HGNC:16993]
ENSG00000067177	PHKA1	2,710093	phosphorylase kinase regulatory subunit alpha 1 [Source:HGNC Symbol;Acc:HGNC:8925]
ENSG00000012232	EXTL3	2,708249	exostosin like glycosyltransferase 3 [Source:HGNC Symbol;Acc:HGNC:3518]

**Biological theme:**	**Protein kinase binding**
**Gene sets:**	Kinase binding (GO)
	Protein kinase binding (GO)

**Ensembl gene ID**	**Gene**	**Weighted mean *z* -score**	**Description**
ENSG00000077044	DGKD	4,182944	diacylglycerol kinase delta [Source:HGNC Symbol;Acc:HGNC:2851]
ENSG00000171206	TRIM8	4,173677	tripartite motif containing 8 [Source:HGNC Symbol;Acc:HGNC:15579]
ENSG00000186591	UBE2H	4,130638	ubiquitin conjugating enzyme E2 H [Source:HGNC Symbol;Acc:HGNC:12484]
ENSG00000110422	HIPK3	4,000689	homeodomain interacting protein kinase 3 [Source:HGNC Symbol;Acc:HGNC:4915]
ENSG00000072062	PRKACA	3,97212	protein kinase cAMP-activated catalytic subunit alpha [Source:HGNC Symbol;Acc:HGNC:9380]
ENSG00000076928	ARHGEF1	3,879147	Rho guanine nucleotide exchange factor 1 [Source:HGNC Symbol;Acc:HGNC:681]
ENSG00000182957	SPATA13	3,82123	spermatogenesis associated 13 [Source:HGNC Symbol;Acc:HGNC:23222]
ENSG00000081320	STK17B	3,755434	serine/threonine kinase 17b [Source:HGNC Symbol;Acc:HGNC:11396]
ENSG00000099875	MKNK2	3,622718	MAP kinase interacting serine/threonine kinase 2 [Source:HGNC Symbol;Acc:HGNC:7111]
ENSG00000141582	CBX4	3,621299	chromobox 4 [Source:HGNC Symbol;Acc:HGNC:1554]
ENSG00000136490	LIMD2	3,572124	LIM domain containing 2 [Source:HGNC Symbol;Acc:HGNC:28142]
ENSG00000063245	EPN1	3,57207	epsin 1 [Source:HGNC Symbol;Acc:HGNC:21604]
ENSG00000129355	CDKN2D	3,567217	cyclin dependent kinase inhibitor 2D [Source:HGNC Symbol;Acc:HGNC:1790]
ENSG00000153179	RASSF3	3,56435	Ras association domain family member 3 [Source:HGNC Symbol;Acc:HGNC:14271]
ENSG00000116604	MEF2D	3,563115	myocyte enhancer factor 2D [Source:HGNC Symbol;Acc:HGNC:6997]
ENSG00000141298	SSH2	3,519703	slingshot protein phosphatase 2 [Source:HGNC Symbol;Acc:HGNC:30580]
ENSG00000177666	PNPLA2	3,505861	patatin like phospholipase domain containing 2 [Source:HGNC Symbol;Acc:HGNC:30802]
ENSG00000185236	RAB11B	3,476148	RAB11B, member RAS oncogene family [Source:HGNC Symbol;Acc:HGNC:9761]
ENSG00000175215	CTDSP2	3,470724	CTD small phosphatase 2 [Source:HGNC Symbol;Acc:HGNC:17077]
ENSG00000086062	B4GALT1	3,465897	beta-1,4-galactosyltransferase 1 [Source:HGNC Symbol;Acc:HGNC:924]
ENSG00000132819	RBM38	3,456865	RNA binding motif protein 38 [Source:HGNC Symbol;Acc:HGNC:15818]
ENSG00000152601	MBNL1	3,439108	muscleblind like splicing regulator 1 [Source:HGNC Symbol;Acc:HGNC:6923]
ENSG00000144579	CTDSP1	3,436199	CTD small phosphatase 1 [Source:HGNC Symbol;Acc:HGNC:21614]
ENSG00000110075	PPP6R3	3,427792	protein phosphatase 6 regulatory subunit 3 [Source:HGNC Symbol;Acc:HGNC:1173]
ENSG00000104915	STX10	3,414787	syntaxin 10 [Source:HGNC Symbol;Acc:HGNC:11428]
ENSG00000137185	ZSCAN9	-3,40946	zinc finger and SCAN domain containing 9 [Source:HGNC Symbol;Acc:HGNC:12984]
ENSG00000157933	SKI	3,376692	SKI proto-oncogene [Source:HGNC Symbol;Acc:HGNC:10896]
ENSG00000078369	GNB1	3,365732	G protein subunit beta 1 [Source:HGNC Symbol;Acc:HGNC:4396]
ENSG00000133818	RRAS2	3,354027	related RAS viral (r-ras) oncogene homolog 2 [Source:HGNC Symbol;Acc:HGNC:17271]
ENSG00000175115	PACS1	3,34959	phosphofurin acidic cluster sorting protein 1 [Source:HGNC Symbol;Acc:HGNC:30032]
ENSG00000184640	SEPT9	3,344101	septin 9 [Source:HGNC Symbol;Acc:HGNC:7323]
ENSG00000127663	KDM4B	3,282037	lysine demethylase 4B [Source:HGNC Symbol;Acc:HGNC:29136]
ENSG00000159840	ZYX	3,277177	zyxin [Source:HGNC Symbol;Acc:HGNC:13200]
ENSG00000112658	SRF	3,251479	serum response factor [Source:HGNC Symbol;Acc:HGNC:11291]
ENSG00000153487	ING1	3,246105	inhibitor of growth family member 1 [Source:HGNC Symbol;Acc:HGNC:6062]

ENSG00000168488	ATXN2L	3,230865	ataxin 2 like [Source:HGNC Symbol;Acc:HGNC:31326]
ENSG00000173120	KDM2A	3,225902	lysine demethylase 2A [Source:HGNC Symbol;Acc:HGNC:13606]
ENSG00000171843	MLLT3	3,218543	MLLT3, super elongation complex subunit [Source:HGNC Symbol;Acc:HGNC:7136]
ENSG00000141522	ARHGDIA	3,215317	Rho GDP dissociation inhibitor alpha [Source:HGNC Symbol;Acc:HGNC:678]
ENSG00000173757	STAT5B	3,195358	signal transducer and activator of transcription 5B [Source:HGNC Symbol;Acc:HGNC:11367]
ENSG00000134954	ETS1	3,166743	ETS proto-oncogene 1, transcription factor [Source:HGNC Symbol;Acc:HGNC:3488]
ENSG00000145349	CAMK2D	3,161391	calcium/calmodulin dependent protein kinase II delta [Source:HGNC Symbol;Acc:HGNC:1462]
ENSG00000103495	MAZ	3,161383	MYC associated zinc finger protein [Source:HGNC Symbol;Acc:HGNC:6914]
ENSG00000171988	JMJD1C	3,151277	jumonji domain containing 1C [Source:HGNC Symbol;Acc:HGNC:12313]
ENSG00000123143	PKN1	3,148454	protein kinase N1 [Source:HGNC Symbol;Acc:HGNC:9405]
ENSG00000136827	TOR1A	-3,1354	torsin family 1 member A [Source:HGNC Symbol;Acc:HGNC:3098]
ENSG00000139974	SLC38A6	-3,12454	solute carrier family 38 member 6 [Source:HGNC Symbol;Acc:HGNC:19863]
ENSG00000125817	CENPB	3,120376	centromere protein B [Source:HGNC Symbol;Acc:HGNC:1852]
ENSG00000138443	ABI2	3,120034	abl interactor 2 [Source:HGNC Symbol;Acc:HGNC:24011]
ENSG00000159335	PTMS	3,104089	parathymosin [Source:HGNC Symbol;Acc:HGNC:9629]
ENSG00000173442	EHBP1L1	3,100877	EH domain binding protein 1 like 1 [Source:HGNC Symbol;Acc:HGNC:30682]
ENSG00000143753	DEGS1	-3,10007	delta 4-desaturase, sphingolipid 1 [Source:HGNC Symbol;Acc:HGNC:13709]
ENSG00000123353	ORMDL2	-3,09818	ORMDL sphingolipid biosynthesis regulator 2 [Source:HGNC Symbol;Acc:HGNC:16037]
ENSG00000135766	EGLN1	3,091759	egl-9 family hypoxia inducible factor 1 [Source:HGNC Symbol;Acc:HGNC:1232]
ENSG00000177169	ULK1	3,091604	unc-51 like autophagy activating kinase 1 [Source:HGNC Symbol;Acc:HGNC:12558]
ENSG00000162889	MAPKAPK2	3,075222	mitogen-activated protein kinase-activated protein kinase 2 [Source:HGNC Symbol;Acc:HGNC:6887]
ENSG00000126458	RRAS	3,070637	related RAS viral (r-ras) oncogene homolog [Source:HGNC Symbol;Acc:HGNC:10447]
ENSG00000196588	MKL1	3,068135	megakaryoblastic leukemia (translocation) 1 [Source:HGNC Symbol;Acc:HGNC:14334]
ENSG00000105723	GSK3A	3,057546	glycogen synthase kinase 3 alpha [Source:HGNC Symbol;Acc:HGNC:4616]
ENSG00000196961	AP2A1	3,054822	adaptor related protein complex 2 alpha 1 subunit [Source:HGNC Symbol;Acc:HGNC:561]
ENSG00000198408	MGEA5	3,054431	meningioma expressed antigen 5 (hyaluronidase) [Source:HGNC Symbol;Acc:HGNC:7056]
ENSG00000177663	IL17RA	3,044475	interleukin 17 receptor A [Source:HGNC Symbol;Acc:HGNC:5985]
ENSG00000155897	ADCY8	-3,01478	adenylate cyclase 8 [Source:HGNC Symbol;Acc:HGNC:239]
ENSG00000047617	ANO2	-3,00797	anoctamin 2 [Source:HGNC Symbol;Acc:HGNC:1183]
ENSG00000160741	CRTC2	3,005593	CREB regulated transcription coactivator 2 [Source:HGNC Symbol;Acc:HGNC:27301]
ENSG00000064666	CNN2	2,993099	calponin 2 [Source:HGNC Symbol;Acc:HGNC:2156]
ENSG00000005483	KMT2E	2,988177	lysine methyltransferase 2E [Source:HGNC Symbol;Acc:HGNC:18541]
ENSG00000215790	SLC35E2	2,977497	solute carrier family 35 member E2 [Source:HGNC Symbol;Acc:HGNC:20863]
ENSG00000155324	GRAMD3	2,976258	GRAM domain containing 3 [Source:HGNC Symbol;Acc:HGNC:24911]
ENSG00000153561	RMND5A	2,973602	required for meiotic nuclear division 5 homolog A [Source:HGNC Symbol;Acc:HGNC:25850]
ENSG00000146700	SSC4D	2,971428	scavenger receptor cysteine rich family member with 4 domains [Source:HGNC Symbol;Acc:HGNC:14461]
ENSG00000142599	RERE	2,968883	arginine-glutamic acid dipeptide repeats [Source:HGNC Symbol;Acc:HGNC:9965]
ENSG00000184922	FMNL1	2,968801	formin like 1 [Source:HGNC Symbol;Acc:HGNC:1212]
ENSG00000253250	C8orf88	2,956469	chromosome 8 open reading frame 88 [Source:HGNC Symbol;Acc:HGNC:44672]
ENSG00000116514	RNF19B	2,952826	ring finger protein 19B [Source:HGNC Symbol;Acc:HGNC:26886]

ENSG00000162620	LRRIQ3	-2,95114	leucine rich repeats and IQ motif containing 3 [Source:HGNC Symbol;Acc:HGNC:28318]
ENSG00000103126	AXIN1	2,950765	axin 1 [Source:HGNC Symbol;Acc:HGNC:903]
ENSG00000228106		-2,94891	
ENSG00000132471	WBP2	2,944219	WW domain binding protein 2 [Source:HGNC Symbol;Acc:HGNC:12738]
ENSG00000171148	TADA3	2,930497	transcriptional adaptor 3 [Source:HGNC Symbol;Acc:HGNC:19422]
ENSG00000180448	ARHGAP45	2,92551	Rho GTPase activating protein 45 [Source:HGNC Symbol;Acc:HGNC:17102]
ENSG00000198909	MAP3K3	2,924523	mitogen-activated protein kinase kinase kinase 3 [Source:HGNC Symbol;Acc:HGNC:6855]
ENSG00000002834	LASP1	2,918575	LIM and SH3 protein 1 [Source:HGNC Symbol;Acc:HGNC:6513]
ENSG00000154229	PRKCA	2,908846	protein kinase C alpha [Source:HGNC Symbol;Acc:HGNC:9393]
ENSG00000167566	NCKAP5L	2,907165	NCK associated protein 5 like [Source:HGNC Symbol;Acc:HGNC:29321]
ENSG00000113384	GOLPH3	2,901079	golgi phosphoprotein 3 [Source:HGNC Symbol;Acc:HGNC:15452]
ENSG00000138764	CCNG2	2,89792	cyclin G2 [Source:HGNC Symbol;Acc:HGNC:1593]
ENSG00000158195	WASF2	2,895007	WAS protein family member 2 [Source:HGNC Symbol;Acc:HGNC:12733]
ENSG00000185652	NTF3	-2,89021	neurotrophin 3 [Source:HGNC Symbol;Acc:HGNC:8023]
ENSG00000131504	DIAPH1	2,888454	diaphanous related formin 1 [Source:HGNC Symbol;Acc:HGNC:2876]
ENSG00000153048	CARHSP1	2,886378	calcium regulated heat stable protein 1 [Source:HGNC Symbol;Acc:HGNC:17150]
ENSG00000006062	MAP3K14	2,881767	mitogen-activated protein kinase kinase kinase 14 [Source:HGNC Symbol;Acc:HGNC:6853]
ENSG00000170891	CYTL1	-2,86633	cytokine like 1 [Source:HGNC Symbol;Acc:HGNC:24435]
ENSG00000133816	MICAL2	2,864662	microtubule associated monooxygenase, calponin and LIM domain containing 2 [Source:HGNC Symbol;Acc:HGNC:24693]
ENSG00000247746	USP51	-2,86454	ubiquitin specific peptidase 51 [Source:HGNC Symbol;Acc:HGNC:23086]
ENSG00000139722	VPS37B	2,860594	VPS37B, ESCRT-I subunit [Source:HGNC Symbol;Acc:HGNC:25754]
ENSG00000198890	PRMT6	-2,85845	protein arginine methyltransferase 6 [Source:HGNC Symbol;Acc:HGNC:18241]
ENSG00000141232	TOB1	2,858099	transducer of ERBB2, 1 [Source:HGNC Symbol;Acc:HGNC:11979]
ENSG00000166501	PRKCB	2,857352	protein kinase C beta [Source:HGNC Symbol;Acc:HGNC:9395]
ENSG00000143437	ARNT	2,854103	aryl hydrocarbon receptor nuclear translocator [Source:HGNC Symbol;Acc:HGNC:700]
